# Simulating within-vector generation of the malaria parasite diversity

**DOI:** 10.1371/journal.pone.0177941

**Published:** 2017-05-22

**Authors:** Lauren M. Childs, Olivia F. Prosper

**Affiliations:** 1 Department of Mathematics, Virginia Tech, Blacksburg, VA, United States of America; 2 Department of Mathematics, University of Kentucky, Lexington, KY, United States of America; Institut national de la santé et de la recherche médicale - Institut Cochin, FRANCE

## Abstract

*Plasmodium falciparum*, the most virulent human malaria parasite, undergoes asexual reproduction within the human host, but reproduces sexually within its vector host, the *Anopheles* mosquito. Consequently, the mosquito stage of the parasite life cycle provides an opportunity to create genetically novel parasites in multiply-infected mosquitoes, potentially increasing parasite population diversity. Despite the important implications for disease transmission and malaria control, a quantitative mapping of how parasite diversity entering a mosquito relates to diversity of the parasite exiting, has not been undertaken. To examine the role that vector biology plays in modulating parasite diversity, we develop a two-part model framework that estimates the diversity as a consequence of different bottlenecks and expansion events occurring during the vector-stage of the parasite life cycle. For the underlying framework, we develop the first stochastic model of within-vector *P. falciparum* parasite dynamics and go on to simulate the dynamics of two parasite subpopulations, emulating multiply infected mosquitoes. We show that incorporating stochasticity is essential to capture the extensive variation in parasite dynamics, particularly in the presence of multiple parasites. In particular, unlike deterministic models, which always predict the most fit parasites to produce the most sporozoites, we find that occasionally only parasites with lower fitness survive to the sporozoite stage. This has important implications for onward transmission. The second part of our framework includes a model of sequence diversity generation resulting from recombination and reassortment between parasites within a mosquito. Our two-part model framework shows that bottlenecks entering the oocyst stage decrease parasite diversity from what is present in the initial gametocyte population in a mosquito’s blood meal. However, diversity increases with the possibility for recombination and proliferation in the formation of sporozoites. Furthermore, when we begin with two parasite subpopulations in the initial gametocyte population, the probability of transmitting more than two unique parasites from mosquito to human is over 50% for a wide range of initial gametocyte densities.

## Introduction

Each year nearly 200 million people are infected with malaria causing parasites, resulting in over half a million deaths, mostly children under the age of five in sub-Saharan Africa [1]. The most virulent species, *Plasmodium falciparum*, exhibits significant diversity in the circulating parasite population [2] even in low endemicity settings [3]. The high level of diversity exhibited across the *P. falciparum* parasite population is unexpected due to the severe bottlenecks during transition between the vertebrate host and the mosquito vector. Furthermore, *P. falciparum* only has capacity to increase diversity via mutation throughout most of its life cycle due to its haploid state, particularly during the longest stage of parasite development, i.e. replication in human red blood cells. Only for a brief period in the mosquito, upon mating, do the parasites form a diploid state [4]. During this period, if multiple parasite populations are present, novel parasites can arise through the process of meiosis.

Mathematical modeling has been a critical component of malaria research. The majority of models have focused on transmission of parasites (e.g. [5–8]) or dynamics within the human host, specifically in the human red blood cells (e.g. [9–14]). The only works focused on parasite dynamics in the mosquito vector consider a genetically identical malaria parasite population and used deterministic transitions between stages in the life cycle [15, 16]. The progression of the life cycle within the mosquito, however, is much more varied with a bottleneck at the oocyst stage [17]. Parasites enter the mosquito through an infectious blood meal, consisting of a few *μ*L of blood [18] which typically contains less than 100 gametocytes (the sexual stage of the parasite able to survive within the mosquito), although up to several thousand have been observed [19–22]. Once gametocytes enter the midgut of the mosquito and experience the change in environment such as a drop in temperature and rise in pH, the parasites begin gametogenesis, the transformation into gametes. In a matter of minutes, the male gametes divide three times to form up to eight motile microgametes while the female gametes mature into single stationary macrogametes. Microgametes search for macrogametes, and upon finding one, fuse to form a diploid zygote which eventually becomes a tetraploid ookinete [23]. The ookinetes migrate through the wall of the midgut and establish themselves as oocysts. Prior to settling as oocysts, they undergo meiosis [24, 25]. As oocysts mature, the parasites undergo 10—11 rounds of endomitosis [26] after which they burst releasing sporozoites into the surrounding extracellular space. The sporozoites must migrate to the salivary glands before they can be transferred to new hosts during an infectious bite. When an infectious mosquito bites a vertebrate host, typically 10-100 sporozoites are transferred into the skin of the new host [27].

A key complication in the understanding of population-level parasite diversity is multiply infected individuals. In fact, many malaria infections of humans, across various transmission settings, appear to contain multiple parasites [28–31]. Quantifying the diversity of parasites within individual infections is challenging as there is a lack of sensitive genetic markers to easily distinguish genetically similar parasites [32, 33]. Multiple parasites can be co-transmitted to a mosquito in a single infectious bite allowing for crossing of parasites in the mosquito midgut [33, 34]. Mixed infections, those harboring multiple distinct parasites, have been suggested to be more infectious [35, 36], despite observations to the contrary [37]. Although it has been generally accepted that recombination within the mosquito increases parasite diversity, there has been little analysis of how diversity generation proceeds mechanistically and its implications for genetic diversity of the population as a whole [38, 39].

In this paper, we address this knowledge gap concerning the mechanistic generation of diversity in the mosquito. To do so, we develop a modeling framework tracking malaria parasite diversity through the various parasite life cycle stages in the mosquito host. As part of this framework, we introduce the first stochastic model of the development of the malaria parasite within the vector. We show that incorporating variation in the dynamics of life stages of the malaria parasite, *Plasmodium falciparum*, within the mosquito leads to a range of sporozoite levels within the salivary glands, with important implications for the capacity for onward transmission. To complete our framework, we couple our stochastic model of parasite number in the mosquito to a stochastic model of sequence diversity generation including recombination between parasites within the mosquito. From our simulations, we find that sequence diversity decreases from entrance of gametocytes in a blood meal to formation of oocysts but the number of unique sequences present in the sporozoite population found in the salivary glands is greatly increased. Those novel sequences are predicted to be frequently passed onto new infections.

## Models

We develop a two-part modeling framework tracking malaria parasite diversity in the mosquito. The first part of this framework concerns the parasite life cycle dynamics in the mosquito, which we implement in two different ways (deterministic and stochastic). In the second part of our modeling framework, we build a stochastic model of sequence diversity generation. This model includes reassortment and recombination of the parasite genome during meiosis. When multiple genetically distinct (defined by at least one difference in a sequence position) parasite populations, are in the same mosquito, new genetically novel parasites can be generated. This model tracks the diversity of parasites within a mosquito through the various life stages in the mosquito, requiring output from the first part of the modeling framework.

### Underlying life cycle model

#### Deterministic implementation

Teboh-Ewungkem and co-authors developed the first within-vector models of *P. falciparum* parasite dynamics in two similar formulations published in 2010 [15, 16]. Here, we utilize a hybrid of the two model formulations, which will serve as the deterministic comparison for our stochastic model developed in the next section. We briefly review the modified version of the Teboh-Ewungkem model below.

The parasite dynamics begin with an initial number of male and female gametes, determined by (1) the initial number *G*_0_ of gametocytes in a bloodmeal, (2) the fraction *m* of gametocytes that are male, (3) the probabilities *α* and *β* that male and female gametocytes successfully undergo gametogenesis to produce gametes, and (4) the average numbers *ρ* and *ν* of gametes resulting from one male or female gametocyte, respectively. Thus, the initial male, *M*_0_, and female, *F*_0_, gamete numbers, are given by:

$$\begin{array}{ccc} M_{0} & = & m\rho \alpha G_{0}\\ F_{0} & = & \left(1-m\right)\nu \beta G_{0} \end{array}$$

The male and female gametes decay due to natural mortality at rates *a* and *b*, respectively. Otherwise, fusion of female *F* and male *M* gametes occurs at a fertilization rate *r* to produce new zygotes *Z*. These zygotes either die at a rate *μ*_*z*_, or transform into ookinetes *E* at a rate *σ*_*z*_. Subsequently, ookinetes will either die at a rate *μ*_*e*_, or transform into oocysts *O* at a rate *σ*_*e*_. Once established, oocysts undergo sporoblast formation, a critical stage in the parasite life-cycle, eventually releasing thousands of sporozoites when they rupture. Continuing with the notation of Teboh-Ewungkem *et al.*, we denote the oocyst mortality rate, the oocyst rupture rate, and the average number of sporozoites produced per oocyst by *μ*_*o*_, *k*(*t*), and *n*_0_ (denoted by *n* in [15, 16]), respectively. Because oocysts only rupture following sporoblast formation, the resulting delay in sporozoite release is captured by a step function, as in [15, 16]:

$$\begin{array}{c} k\left(t\right)=\{\begin{array}{cc} 0, & \;\text{if}\;0<t<t_{0}\\ d, & \;\text{if}\;t\geq t_{0} \end{array} \end{array}$$

where *t*_0_ is the time until mature oocysts have formed, and *d* is the rate at which mature oocysts release sporozoites. Finally, only a fraction *p* of sporozoites will successfully migrate to, and invade, the salivary glands producing *S* sporozoites found there. The full deterministic model is described by the following system of non-autonomous, ordinary differential equations [15, 16], and all parameters are found in Table 1.

$$\begin{array}{ccc} M^{\prime } & = & -aM-rMF\\ F^{\prime } & = & -bF-rMF\\ Z^{\prime } & = & rMF-(\sigma_{z}+\mu_{z})Z\\ E^{\prime } & = & \sigma_{z}Z-\left(\sigma_{e}+\mu_{e}\right)E\\ O^{\prime } & = & \sigma_{e}E-\left(k\left(t\right)+\mu_{o}\right)O\\ S^{\prime } & = & n_{0}pk\left(t\right)O. \end{array}$$

**Table 1:** Parameter values for the underlying within-vector model. All values, apart from *G*_0_ and *d*, were obtained from Teboh-Ewungkem *et al.* [15]. *G*_0_ was varied. *d* was obtained from Teboh-Ewungkem *et al.* [16].

Symbol	Description	Default value
*G*_0_	number of gametocytes in blood meal	{150, 200, …, 450}
*m*	percentage of gametocytes that are male	0.25
*ν*	number of female gametes per female gametocyte	1
*ρ*	number of male gametes per male gametocytes	8
*α*	fraction of male gametes that are viable	0.39
*β*	fraction of female gametes that are viable	1
*a*	failure rate of male gametocytes (per day)	24/(20/60)
*b*	failure rate of female gametocytes (per day)	24/(25/60)
*r*	fertilization of male and female gametes (per parasite per day)	0.08
*μ*_*z*_	death rate of zygotes (per day)	1
*μ*_*e*_	death rate of ookinetes (per day)	1.4
*μ*_*o*_	death rate of oocysts (per day)	0
*σ*_*z*_	transformation rate of zygotes (per day)	24/19
*σ*_*e*_	transformation rate of ookinetes (per day)	0.6
*n*_0_	number of sporozoites per oocyst	3000
*p*	proportion of sporozoites that make it to salivary gland	0.2
*t*_0_	time to mature oocyst formation (days)	10
*d*	rate mature oocysts release sporozoites (per day)	$\frac{1}{7}$

#### Stochastic implementation

To better evaluate within-vector parasite variation, we developed a continuous time, discrete state Markov Chain (CTMC) extension of the Teboh-Ewungkem models (see S1 Fig).

In this stochastic model, we assumed there exists a time interval Δ*t* small enough that at most one event in the parasite dynamics can occur within this window. These events include deaths from each stage of the within-vector parasite life-cycle and progressions from one stage to the next. The simulations were carried out using a small modification to the traditional Gillespie Algorithm, for which the inter-event times are exponentially distributed, with probabilities and transitions described in Table 2 and the same initial conditions as in the deterministic model (see S1 Appendix for an outline of the algorithm). The step-function formulation for the oocyst bursting rate *k*(*t*), introduced by Teboh-Ewungkem *et al.* and stated above in the deterministic formulation of the model, assumes all oocysts reach maturity at the same time. To be more biologically accurate, we relax this assumption and introduce a continuous time function with a similar profile: *β*(*t*) = (1 + exp(*t*_0_ − *t*))^−1^, which is small for small values of *t*, 0.5 when *t* = *t*_0_, and approaches one as *t* approaches infinity. Recall that *d* is the rupture rate of mature oocysts. Thus, the bursting rate function in our stochastic model is *k*(*t*) = *dβ*(*t*).

**Table 2:** CTMC transition probabilities for the stochastic within-vector model. *Note that probabilities are denoted in the standard form used by [40]. For calculation, the term as written is divided by the sum of all transitions. See SI File for details.

Event	Probabilities*	Transitions
Death of Male gamete	*aM*Δ*t* + *o*(Δ*t*)	(−1, 0, 0, 0, 0, 0)
Mating	*rMF*Δ*t* + *o*(Δ*t*)	(−1, −1, 1, 0, 0, 0)
Death of Female gamete	*bF*Δ*t* + *o*(Δ*t*)	(0, −1, 0, 0, 0, 0)
Death of Zygote	*μ*_*z*_*Z*Δ*t* + *o*(Δ*t*)	(0, 0, −1, 0, 0, 0)
Zygote to Ookinete progression	*σ*_*z*_*Z*Δ*t* + *o*(Δ*t*)	(0, 0, −1, 1, 0, 0)
Death of Ookinete	*μ*_*e*_*E*Δ*t* + *o*(Δ*t*)	(0, 0, 0, −1, 0, 0)
Ookinete to Oocyst progression	*σ*_*e*_*E*Δ*t* + *o*(Δ*t*)	(0, 0, 0, −1, 1, 0)
Death of Oocyst	*μ*_*o*_*O*Δ*t* + *o*(Δ*t*)	(0, 0, 0, 0, −1, 0)
Bursting of Oocyst (sporozoite production)	*k*(*t*)*O*Δ*t* + *o*(Δ*t*)	(0, 0, 0, 0, −1, *n*)

To introduce variability in the number of sporozoites *n* released from one bursting oocyst, we first drew the number of sporozoites per oocyst from a Poisson distribution with mean *n* = *n*_0_. Because only a fraction of sporozoites successfully migrate to the vector salivary glands, we then, independently, selected a portion of these sporozoites from a binomial distribution with probability of success *p*.

#### Multiple genetically distinct parasite populations

Humans are often infected with multiple genetically distinct *Plasmodium* parasite subpopulations [28–30], and consequently, a mosquito blood meal can be composed of multiple genetically distinct parasites. A fitness advantage of different parasites within the same mosquito host has been exemplified experimentally [29]. To this end, we extended the stochastic life cycle model to incorporate *N* genetically distinct parasites with different survival probabilities. We assume that parasites with greater survival probability have a higher fitness. For example, in the case where *N* = 2 (which we consider in depth in this paper), that is, where we modeled two genetically distinct parasite subpopulations within the mosquito, we assumed that parasite 1 is more fit than parasite 2, with the mortality rate in each stage of the life cycle (beginning with the gamete stage) enhanced by 10% (or 50%) for parasite 2, referred to as 10% (or 50%) bias throughout. Four types of zygotes may result from the two genetically distinct parasites: *Z*_1,1_, *Z*_1,2_, *Z*_2,1_, *Z*_2,2_, where *Z*_*i*,*j*_ denotes a zygote forming from the fertilization of a type *i* female gamete by a type *j* male gamete. As we have no knowledge of how the fitness of the progeny of two parasites will be effected, we assume the simplest function, i.e. that the fitness of each progeny is the average of their parents; for example, the mortality rate of *Z*_1,2_ is the average of the mortality rates for *Z*_1,1_ and *Z*_2,2_.

The model keeps track of the number of zygotes, ookinetes, oocysts, and sporozoites resulting from the four possible sets of “parent parasites”. The transition matrix is defined in a similar fashion for the two parasite case as for the single genetically distinct parasite model, only now there are two types of ‘Death of a male gamete’ events: the death of a parasite 1 gamete, and the death of a parasite 2 gamete. For two parasites, there are four possible types of ‘fertilization’ events, resulting in the four types of zygotes discussed earlier. If the event in a particular time-step is the fertilization of a parasite 1 female gamete by a parasite 2 male gamete, then the transition matrix is defined such that the number of parasite 1 female gametes, and the number of parasite 2 male gametes both *decrease* by 1, and the number of parasite 1,2 zygotes (*Z*_1,2_) *increases* by one. We present the complete transition matrix for the two parasite case in S1 Table. All code for the life-cycle model can be found in S1 File.

### Model of diversity generation

We modeled parasite diversity as it changes during the parasite life cycle in the mosquito (see S2 Fig). We represent each parasite by a sequence of length *L* with each position taken from an alphabet of length *A*. We calculated the diversity of the simulated parasite population as entering gametocytes, ruptured oocysts, sporozoites in the salivary glands, and exiting sporozoites during a single mosquito infection. All code for the diversity model can be found in S2 File.

#### Definition of parasite barcode sequence

The genetic basis of this study is supported by the segregation of 24 positions in the genome (*L* = 24) that will be considered as biallelic (*A* = 2), motivated by the 24-position SNP (Single Nucleotide Polymorphism) barcode developed by Daniels *et al.* (2008) to uniquely identify parasite isolates [41]. In other words, we only simulated the barcode SNPs, which in the model appear as an ordered list of 24 positions each with just two possible alleles (0 or 1). For simplicity, the model remains bi-allelic despite evidence that one of the SNPs, SNP #15 (*Pf*_07_001415182), is tri-allelic [42]. These 24 positions are located throughout the 14 *P. falciparum* chromosomes at known points (see S1 Appendix for the exact locations). As the distance between SNPs will effect the likelihood of recombination, we considered the precise position of the barcode SNPs when we formalized recombination (see S1 Appendix for recombination algorithm). It is important to note that diversity, as we calculate it, can only be non-zero when multiple genetically distinct parasite populations are present. Furthermore, for ease of notation, throughout the manuscript we will refer to the simulated sequence of 24 positions representing each unique parasite as its *barcode sequence*.

#### Genetically distinct parasite populations

The starting pool of gametocytes, emulating an infectious blood meal from a human host, consisted of a single barcode sequence for each parasite subpopulation present. In the case of infections consisting of two parasites, the founding parasite subpopulation barcode sequences differed by 0 ≤ *p*_*B*_ ≤ 24 positions. For example, when *N* = 2 there were two genetically distinct parasite populations whose numbers came from from the underlying life cycle model (in either its deterministic or stochastic implementation). Following fertilization between these two distinct parasites, the resulting barcode sequences were either of type *Z*_1,2_ or *Z*_2,1_ (described above in the life cycle model). The order of the subscript (female first, male second) denoted from which parasite population each parent came. The number of parasites in each stage up to the formation of oocysts occurred with loss, the magnitude of which was associated with the fitness bias of the starting parasite.

#### Simulating recombination and reassortment

While the barcode sequences were paired in the oocyst stage, we simulated recombination and reassortment. To simplify the genetic mechanism of this study, during recombination, crossovers were simulated on each chromosome according to a Poisson distribution [43, 44] with mean one [44]. When a crossover occurred, the location of the crossover was chosen uniformly across the length of the chromosome. Multiple crossover events occurred iteratively. Following each crossover event, the resulting individual haploid strands were chosen uniformly for the next crossover event and then the location of the crossover was chosen uniformly across the length of the chromosome. As only the allele at the chromosomal position of the barcode SNP were recorded, not all crossovers changed the barcode sequence.

Following recombination, we simulated reassortment by selecting a single version of each chromosome to be packaged together. When only a single barcode SNP position appeared on a chromosome, whether the recorded allele changed depended solely on reassortment. When multiple barcode SNP positions appeared on the same chromosome, then recombination could separate the recorded alleles, with the probability dependent upon the distance between the SNP positions on the chromosome. In this way, whether or not exchanges of genetic material occurred between the two mating parasites was determined for each of the 14 chromosomes, producing up to four unique barcode sequences per mating event.

#### Measuring diversity

We measured the diversity of the within-vector parasite population in two ways: nucleotide diversity and number of unique barcode sequences. We used a standard measure of nucleotide diversity, *π*, to measure the genetic variation in the within-mosquito population, $\pi =\sum_{ij}\frac{n_{i}n_{j}H_{ij}}{L}$, where *n*_*i*_ and *n*_*j*_ are the frequency of genetically distinct parasite subpopulations *i* and *j*, *H*_*ij*_ is the Hamming distance between the two parasite subpopulations, and *L* is the total number of positions in each sequence. The number of unique barcode sequences that occurred in each mosquito represents a population richness type metric, commonly used in ecology. We chose these metrics to account for similarity to the entering parasite populations (identity by decent) due to recombination (see S1 Appendix for a further discussion of diversity metrics).

Comparisons of the diversity were made at each of the following stages: (1) gametocytes upon entering the mosquito, (2) burst oocysts in the mosquito, (3) sporozoites in the mosquito salivary glands, and (4) sporozoites found in an infectious bite. We assumed an infectious bite contained ten sporozoites randomly chosen from the total pool of salivary gland sporozoites [45].

## Results and discussion

### Comparing the CTMC model to the deterministic model

#### Model of a single genetically distinct parasite population

**Fig 1:**
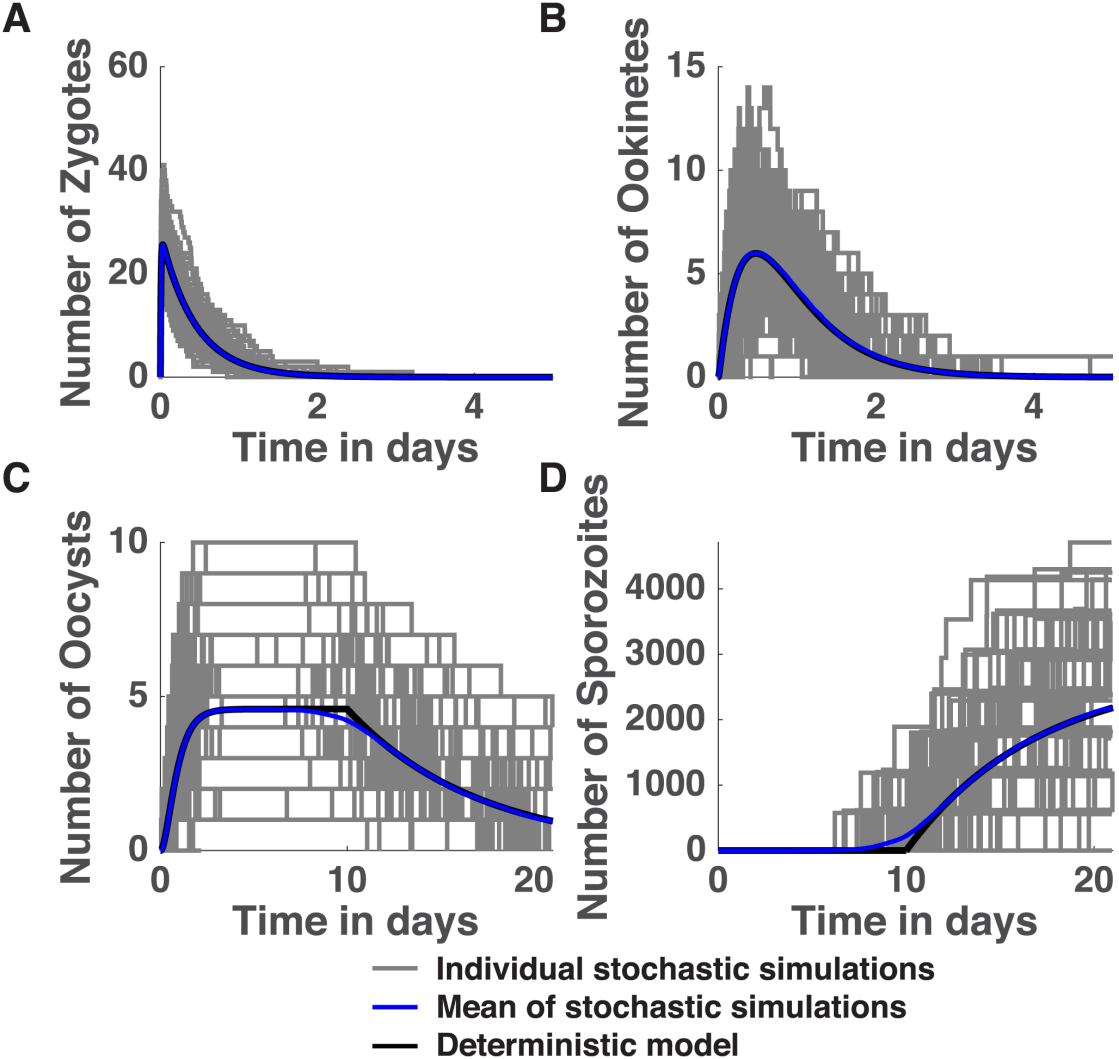
Dynamics of the within-vector model. The temporal dynamics of the deterministic model (black line) and the average dynamics across 1000 stochastic simulations (blue curve) overlays 100 simulations of the single-parasite population CTMC model (gray lines) with initial gametocyte number *G*_0_ = 300 for zygotes (A), ookinetes (B), oocysts (C) and sporozoites (D). Observe that not all simulations produce a positive number of sporozoites. In fact, out of 10,000 simulations, 2.58% produce no sporozoites.

The CTMC model of the parasite life cycle captures the average dynamics generated by the deterministic implementation of the model (Fig 1). However, the CTMC model is also able to capture variation in the life cycle. In the initial parasite stages, namely the gamete and zygote stages, the variation in the dynamics of the stochastic model is relatively small, and therefore the average dynamics captured by the deterministic model provides an accurate approximation to the population dynamics exhibited by its stochastic counterpart. However, as the parasites progress through successive stages, the variation in the stochastic output increases, and the mean captured by the deterministic model becomes less and less representative of the parasite population in any given mosquito. For example, using the baseline parameter values, the deterministic model results in a positive number of parasites at each stage of the within-vector life-cycle. However, 2.58% of the 10,000 stochastic simulations resulted in no sporozoites (Fig 1). The inability of the deterministic model to measure variation in parasite numbers could lead to erroneous estimates of parasite diversity at the population level. Furthermore, the fact that the deterministic model is continuous in state poses a challenge when linked to the sequence diversity model, since fractional oocysts still lead to the production of sporozoites.

We track the parasite dynamics within each mosquito for a time span of 21 days in all model simulations. As expected, sporozoite prevalence in the model, defined as the proportion out of 10,000 simulations resulting in at least one sporozoite in the salivary glands by day 21, increases with increasing initial gametocyte number *G*_0_. In fact, for *G*_0_ = 150, 200, …, 450, the sporozoite prevalence is 62.90%, 82.98%, 92.95%, 97.42%, 99.47%, 99.84%, 99.95%, respectively. In S3 Fig, we show the dependence of oocyst and sporozoite prevalence as a function of *G*_0_ (with *G*_0_ ranging from 10 to 900), on days 7, 14, and 21. On day 14, all simulations with an initial gametocyte number of *G*_0_ = 500 or greater harbor sporozoites in the salivary glands. However, on day 7 of a mosquito infection, most simulated mosquitoes will not be infectious to humans, even with an initial number of gametocytes equal to *G*_0_ = 900.

**Fig 2:**
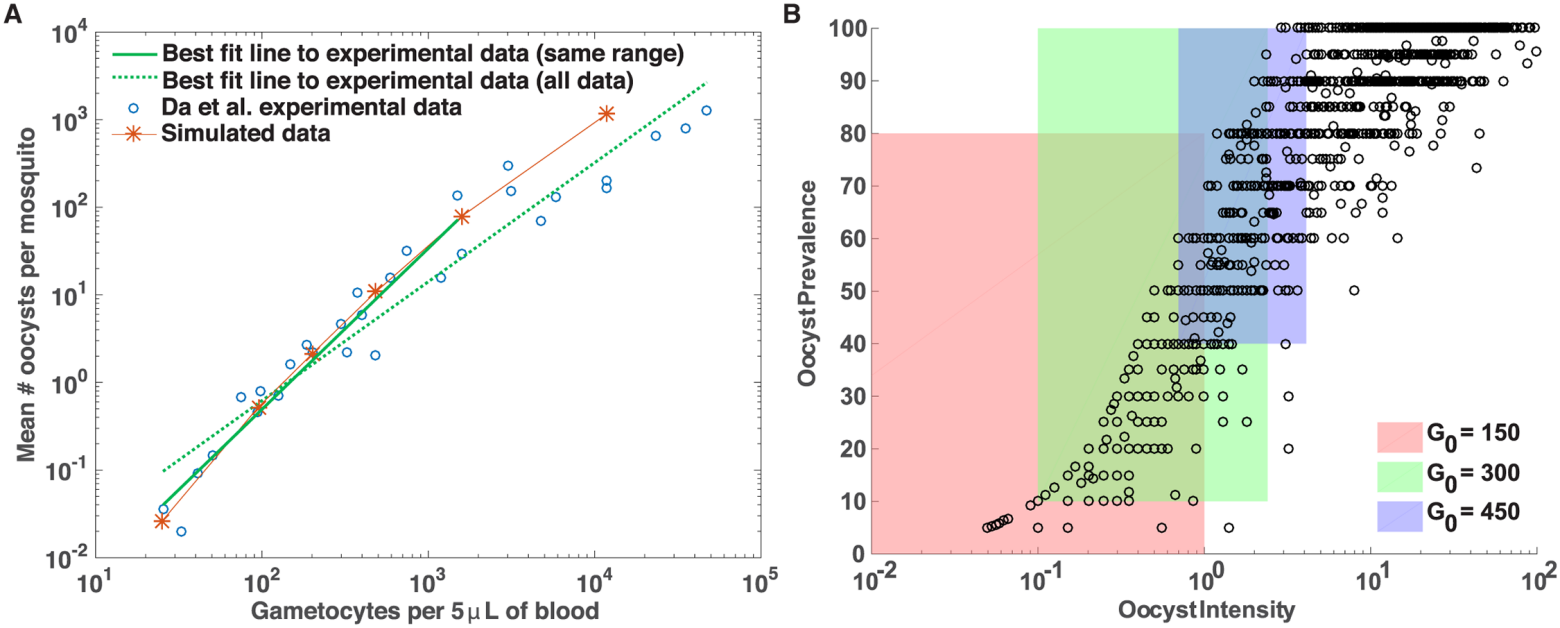
Comparison of life cycle model with published data. (A) Experimental data (blue circles) extracted (using Plot Digitizer) from Figure 1A in [46] along with the mean number of oocysts per mosquito calculated from our simulation data. The experimental data is scaled to be in units of gametocytes per five *μ*L rather than gametocytes per one *μ*L. A regression line to the log-log experimental data (dashed green) and to a subset of the experimental data over the range of initial gametocyte densities used to parameterize the model (solid green) is shown along with our model simulations (red stars). (B) Experimental data (black circles) from Figure 1 in [47] of oocyst intensity and prevalence (reproduced with permission) compared to results of the simulation starting from 150 (red shading), 300 (green shading) and 450 (blue shading) gametocytes. The colored areas represent the range of oocyst intensity and prevalence observed from simulations.

Over the range of initial gametocyte densities that we consider in our model, oocyst densities are similar to reported by Da *et al* [46] suggesting that our model construction and parameterization produces realistic oocyst densities, at least in the initial gametocyte range *G*_0_ ∈ [0, 1000] (see Fig 2A). Da *et al* [46] dissected 1,636 female *Anopheles coluzzi* mosquitoes seven days after feeding them with blood containing gametocyte densities ranging between 80 and 9,520 gametocytes per microliter. For each initial gametocyte density, the mean number of oocysts per mosquito was reported. In our model, we assume a bloodmeal is five *μ*L; thus, *G*_0_ = 450 indicates that there are 450 gametocytes per 5 *μ*L. In Fig 2A, the experimental data is scaled to be in units of gametocytes per five *μ*L. Despite differences in mosquito species (our model assumes parameter values for *Anopheles gambiae*, while the experimental data consists of measurements from *Anopheles coluzzi*), the mean number of oocysts per mosquito as a function of initial gametocyte density per five *μ*L of blood is remarkably consistent between the experimental data in [46] and our simulation data, particularly for *G*_0_ ∈ [0, 1000]. Thus, we have confidence the simulation results presented in S3 Fig are realistic. When the full range of gametocyte densities considered in [46] is included in the regression analysis, the slope of the regression line is smaller, suggesting that the pattern observed for *G*_0_ ∈ [0, 1000] is different for gametocyte densities beyond this range, namely the number of oocysts produced per gametocyte is diminished at very high initial gametocyte densities.

#### Model of two genetically distinct parasite populations

**Fig 3:**
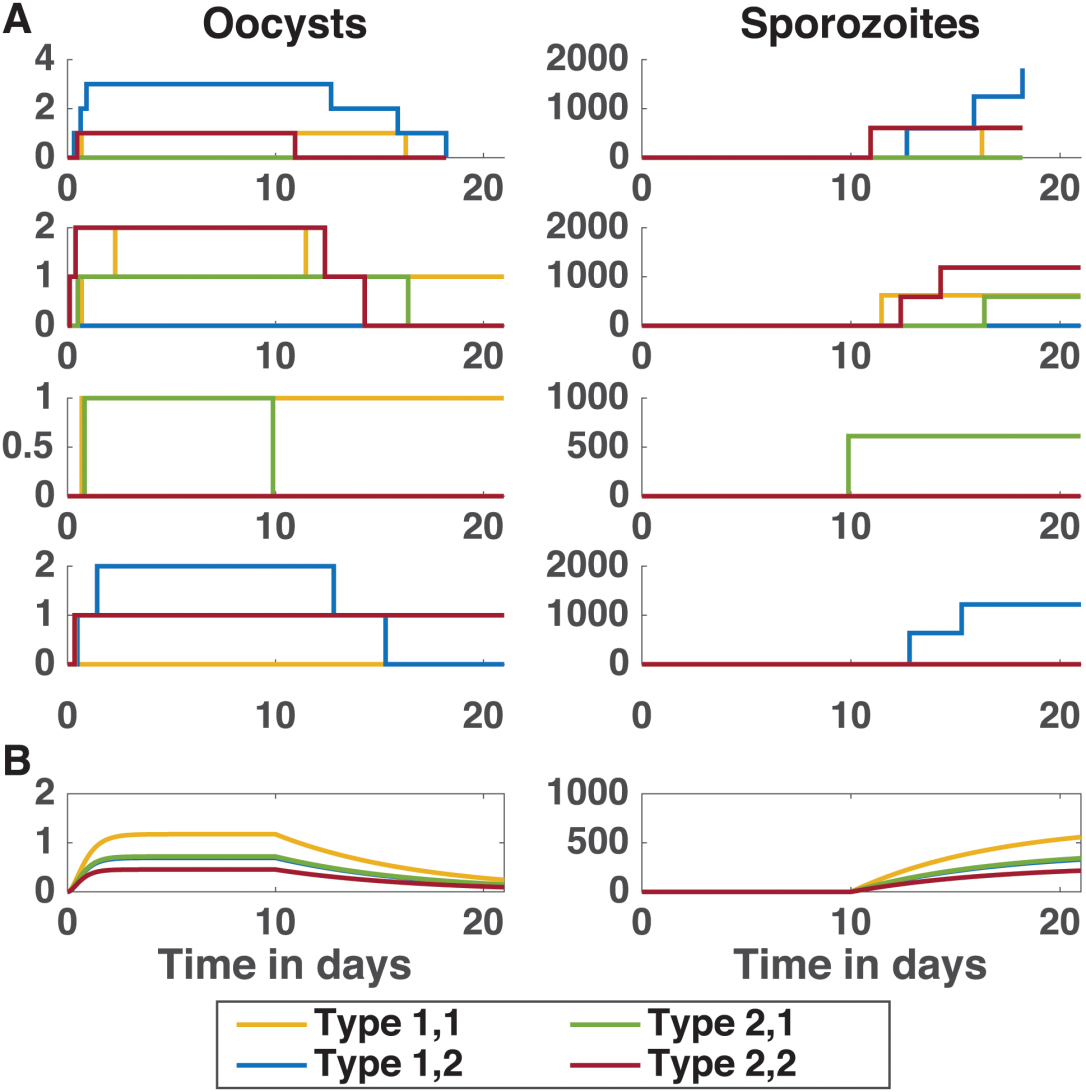
Comparison of stochastic and deterministic dynamics of oocysts and sporozoites. (A) Each of the first four rows illustrate the dynamics of the oocyst (left column) and sporozoite (right column) populations from individual simulations of the CTMC model using 50% fitness bias and *G*_0_ = 300. (B) The dynamics of the deterministic model for oocysts (left) and sporozoites (right). Observe the variation in dynamics possible with the CTMC model.

A range of dynamics are observed for the two parasite population CTMC model (Fig 3A): the order of which subpopulations are most numerous at each change can change while the deterministic model always gives identical ordering (Fig 3B). For example, while the deterministic model always produces a positive number of oocysts and sporozoites, some of the stochastic simulations produce no oocysts, and some of the oocysts that do arise never burst within the 21-day timespan. Furthermore, the deterministic model always results in type 1,1 outperforming type 2,1, followed closely by type 1,2, and finally type 2,2 parasites at each parasite stage. The stochastic model yields the same results on average (taken across all 10,000 simulations), but in contrast to the deterministic model, individual simulations can exhibit a wide range of behavior, as suggested by Fig 3A, including simulations where the least fit parasite type 2,2 is the only one surviving to the sporozoite stage.

**Fig 4:**
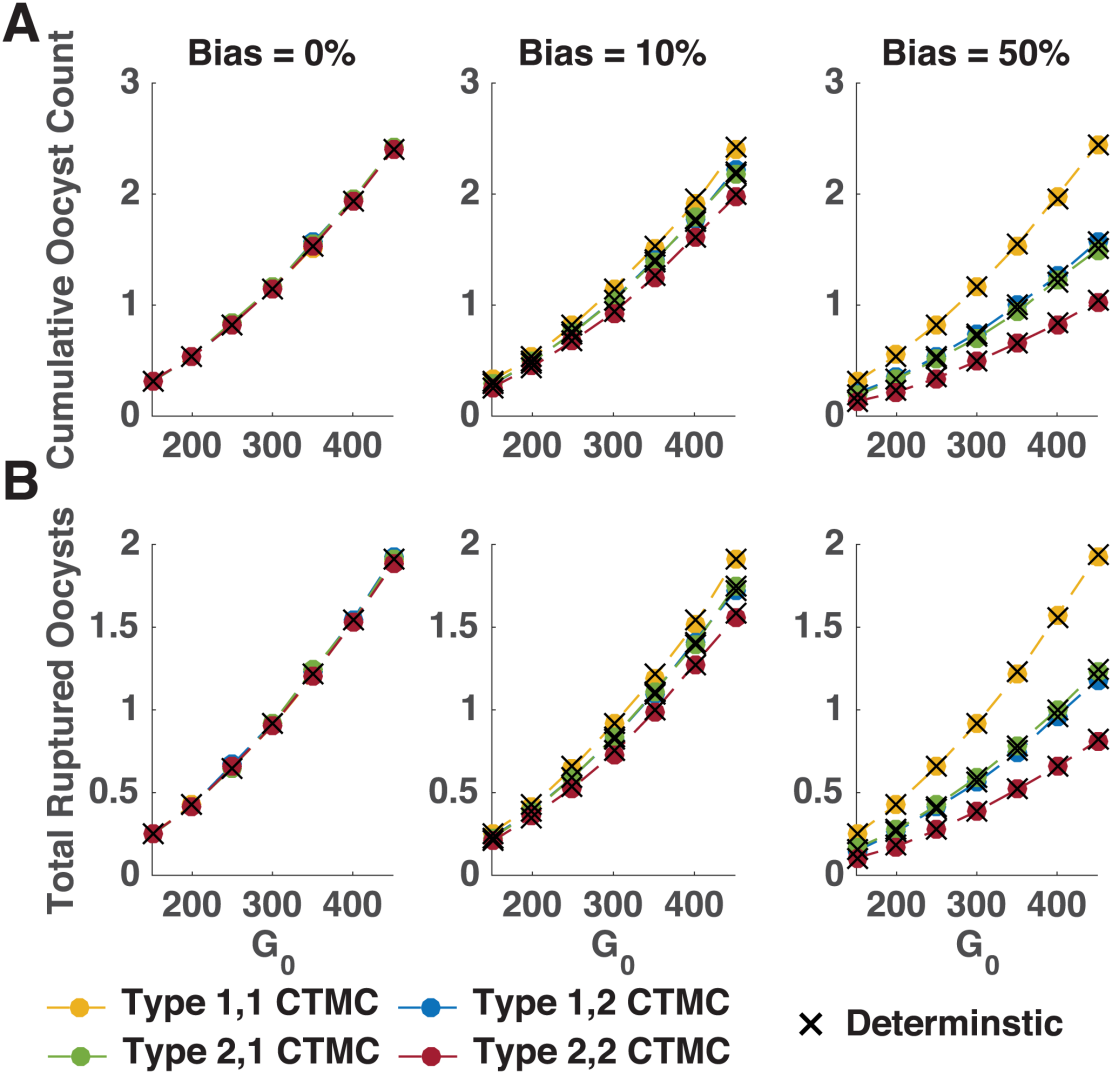
Cumulative and ruptured oocysts. The mean cumulative number (on day 21) of oocysts formed (A) and the total number of ruptured oocysts (B) resulting from the 10,000 simulations of the two-parasite population CTMC model (dots) and deterministic model (crosses) is illustrated for initial gametocyte number *G*_0_. Here, the CTMC model utilizes a more biologically realistic continuous rupture function for oocyst bursting, compared to the step function used in the deterministic model. (See S4 Fig for the comparison of models both using a step bursting function.) Left to right the columns illustrate the results for 0%, 10% and 50% fitness biases, respectively.

While Fig 3 shows some example simulations, when all 10,000 simulations of the CTMC model are combined, the CTMC remains a good approximation of the deterministic model (Fig 4). The average cumulative number of oocysts formed by day 21 (Fig 4A) and the cumulative number of oocysts that rupture by day 21 (Fig 4B) are nearly identical for the stochastic and deterministic models, illustrated by colored circles and black crosses, respectively.

Recall that the deterministic model employs a discontinuous rupture function, which we approximate in the stochastic model by a continuous rupture function to allow some oocysts to rupture before day ten, with low probability. To ensure that differences were not the result of our choice of rupture function, we compared simulations of the stochastic model using the identical discontinuous rupture function. Our results revealed that assuming a continuous rupture function has little impact on cumulative oocyst and rupture numbers on day 21 (see S4 Fig) or the distribution of cumulative oocysts ruptured (see S5 Fig). Despite these similarities, the two types of rupture functions lead to different distributions of rupture timing, with most oocysts rupturing at intermediate values of time under the continuous rupture function while time to oocyst rupture decays with time under the step rupture function (see S6 Fig). This notable difference in the distribution of rupture times, and thus difference in the appearance of sporozoites in the salivary glands, has potentially important implications for onward transmission to a human host, given that female mosquitoes take multiple bloodmeals at different times throughout their life cycle.

**Fig 5:**
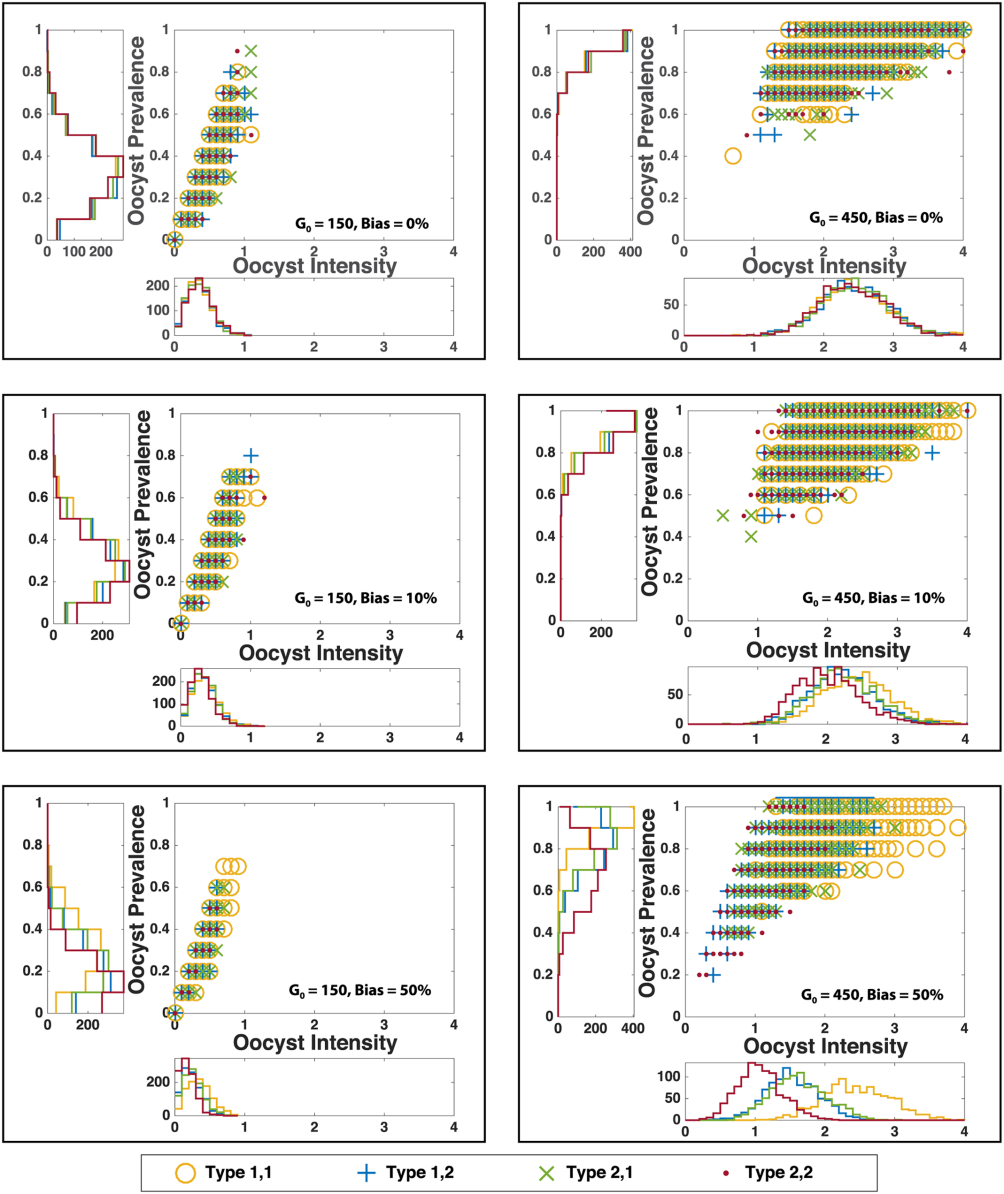
Oocyst intensity and prevalence. The scatter plots show mean oocyst intensity (x-axis) versus mean oocyst prevalence (y-axis), calculated 1000 times. *Oocyst prevalence* is defined as the proportion of mosquitoes harboring oocysts (i.e. simulations producing at least one oocyst). *Oocyst intensity* is defined as the number of oocysts per mosquito (i.e. per simulation). To produce each point, we randomly drew ten mosquitoes (that is, ten stochastic realizations) from our 10,000 simulations of the stochastic model and computed the oocyst prevalence and intensity for these ten mosquitoes on day seven. The histograms show the density of points in the scatter plot for different values of oocyst intensity (horizontal graph) and oocyst prevalence (vertical graph). In the left and right columns, the initial gametocyte number is *G*_0_ = 150 and *G*_0_ = 450, respectively. Top to bottom the rows illustrate the results for 0%, 10% and 50% fitness biases, respectively. Observe that as *G*_0_ increases, the differences in distribution of the genetically distinct parasite populations becomes more pronounced.

We compared our simulations to experimental work conducted by Stone *et al* [47] in which prevalence and intensity of oocysts were measured. *Oocyst prevalence* is defined as the proportion of mosquitoes harboring oocysts (i.e. simulations producing at least one oocyst). *Oocyst intensity* is defined as the number of oocysts per mosquito (i.e. per simulation). Stone *et al.* calculated these quantities experimentally by computing the oocyst prevalence and mean oocyst intensity between days six and nine, post infection, for groups of *A. stephensi* and *A. gambiae* mosquitoes with a sample size greater than ten [47]. They included a total of 21,240 mosquitoes in these calculations. To emulate these experiments, we randomly drew ten mosquitoes (that is, ten stochastic realizations) from our 10,000 simulations of the stochastic model and computed the oocyst prevalence and intensity for these ten mosquitoes on day seven (to be consistent with experimental comparisons [47]). We then repeated this calculation 1000 times to produce the scatter plots and distributions in Fig 5. Consequently, each point in the scatter plot represents an average taken over a group of ten mosquitoes (simulations), and the resulting plots are analogous to Fig 1 in Stone *et al.* [47] (Note: the x-axes of the scatter plots are restricted to the interval [0, 4] for illustrative purposes; the range exceeds [0, 4] in some simulation experiments). Under a 0% bias, approximately 63.0% and 99.9% of simulations resulted in ruptured oocysts, when *G*_0_ = 150 and *G*_0_ = 450, respectively. Under a 10% bias, the prevalence of ruptured oocysts is 60.3% and 99.9%, respectively. Under a 50% bias, the prevalence of ruptured oocysts is 48.6% and 99.5%, respectively.

To facilitate a comparison between our simulation results presented in Fig 5 and the experimental results in Stone et al. [47], we have recreated Figure 1 in [47], with summary results of our simulations overlaying the scatter plot of the experimental data (see Fig 2B). Although Figs 5 and 2B differ quantitatively, the figures are qualitatively similar. Furthermore, our summary statistics and those of the Stone *et al* experiments are surprisingly comparable, particularly when taking into consideration the timespan of the Stone *et al* experiments. The rectangles depicted in Fig 2B illustrate the range of oocyst intensity and oocyst prevalence values obtained from each group of 10 simulated mosquitoes in Fig 5, for initial gametocyte densities *G*_0_ = 150, 300, and 450 and a 10% fitness bias. For example, for *G*_0_ = 300 and a 10% fitness bias, the range in mean oocyst intensity is 0.1 to 2.4, the range in mean oocyst prevalence is 0.1 to 1, and the median number of oocysts across all 10,000 simulated mosquitoes is 1. The range in oocyst numbers across all 10,000 simulated mosquitoes is 1 to 7 for these starting conditions. These summary statistics for each initial gametocyte number and for each fitness bias are presented in S2 Table. For comparison, the Stone *et al.* experiments yielded an infection prevalence, measured at seven days post infection, ranging from 33% to 86.5%, and mean oocyst intensity ranging between 0.57 and 4.7. Furthermore, our simulation results yielded approximately 56%, 41%, and 21%, on average, of oocysts remained intact on days 7, 14, and 21, respectively, whereas the experiments in [47] showed that 25% of oocysts remained intact 21 days post infection, with few oocysts rupturing between day 14 and day 21.

### Diversity results

During the life cycle of the parasite in the mosquito host, there is a severe bottleneck at the oocyst stage with only a handful of parasites able to successfully mate and produce sporozoites. Due to this bottleneck, even with the presence of reassortment and recombination among chromosomes, the sporozoite population would not be expected to have an increased nucleotide diversity compared to the entering gametocyte population, in the absence of new mutations. However, the variation exhibited in the oocyst and sporozoite populations may include newly generated combinations of existing genetic pieces, creating novel variation. Naturally, this variation cannot occur when only a single genetically distinct parasite population is present because any recombination or reassortment would be with the same original barcode sequence.

#### Modeling two genetically distinct parasite populations

**Fig 6:**
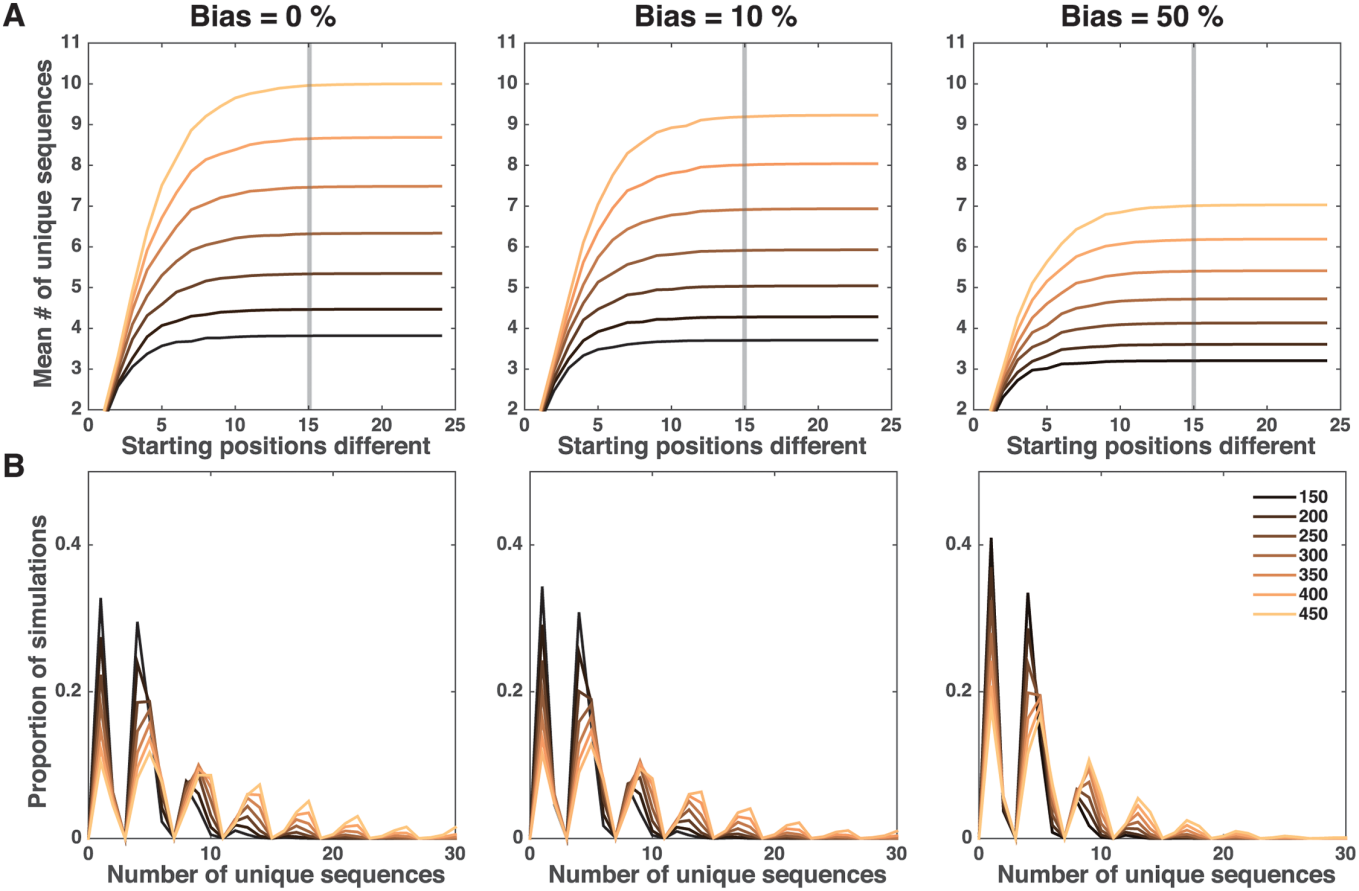
Number of unique barcode sequences. (A) The number of unique barcode sequences from two genetically distinct parasite populations as a function of the number of barcode positions that initially differ. (B) Distribution of the unique barcode sequences when looking at 15 differences between the two initial parasite populations. Colors indicate the number of starting gametocytes with darker colors representing lower initial gametocyte numbers. Left to right the columns illustrate the results for 0%, 10% and 50% fitness biases, respectively. Gray bars in (A) show the similarity in parasite populations for the distributions in (B).

Fig 6 shows an increase in the number of new genetically distinct parasite populations as quantified by the number of unique parasite barcode sequences present at the oocyst stage. New barcode sequences arise through the reassortment and recombination of existing alleles. As the two initial parasite populations decrease in similarity, i.e. the number of differences in barcode sequences (*p*_*B*_) increases, the number of unique barcode sequences rapidly increases before leveling off. The sharp increase is observed until approximately 10 differences exist between the parasite populations. As the initial barcode sequences become more disparate, the possible ways to create new parasite barcode sequences is so great that almost every new barcode sequence generated is unique and the number of unique barcode sequences is limited by the total number of barcode sequences created during oocyst formation. Fig 6B shows that at every initial gametocyte number there is a substantial peak of simulations that harbor only a single genetically distinct parasite population, the result of parasites only mating with their identical siblings. As the initial gametocyte number increases, more of the simulations result in a higher number of unique barcode sequences, i.e. the density of peaks shifts to the right. Because recombination between two parasites generally results in one or four unique barcode sequences (if they are identical or different, respectively) certain numbers of unique barcode sequences are not possible in a single mosquito. For example, it is not possible to obtain three unique sequences in a single mosquito when starting with two parasite subpopulations. Our results are consistent with experimental results, which used microsatellite markers to quantify the parasite diversity within a mosquito. They found fluctuations of genotypes, specific parasite sequences, throughout development and the presence of genotypes in the sporozoite population that were not detected in the gametocyte population [29].

**Fig 7:**
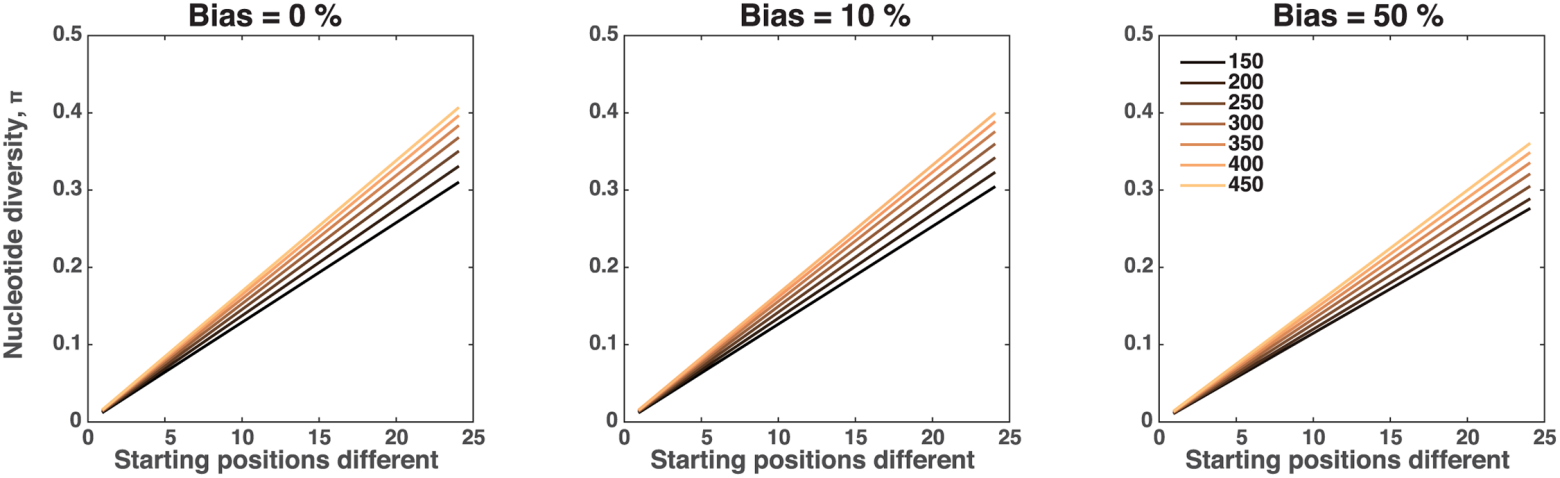
Nucleotide diversity of ruptured oocysts. Nucleotide diversity, *π*, by differing number of starting positions between genetically distinct parasite populations. Left to right the columns illustrate the results for 0%, 10% and 50% fitness biases, respectively. Colors refer to the initial gametocyte number with darker colors representing lower gametocyte numbers.

In Fig 7, the nucleotide diversity increases linearly as the two genetically distinct parasite populations decrease in similarity, as measured by the number of simulated barcode positions which differ. The rate of linear increase, however, depends on the size of the initial gametocyte population (line colors) as well as the fitness bias of one parasite population (0% on the left to 50% on the right). The increase with higher initial gametocyte number is further enhanced as more initial positions differ between the two parasite populations. Comparatively, the strength of the fitness bias only slightly impacts the increase in nucleotide diversity, with greater differences observed when the initial parasite populations are most disparate (Fig 7, right panel).

This decrease in diversity observed in populations where one parasite population has a high fitness bias is due to the decreased survival of each of the life stages for the low fitness population. When the success of one parasite population is severely limited, the potential for diversity in the full population is also limited as no new diversity can be created, either by reassortment or recombination, when a parasite mates with an identical sibling. The increase in nucleotide diversity observed as the number of differing positions increases is due to the reduction in the proportion of the population that exhibits no diversity, i.e. the proportion where only identical sequences are found (see S7 Fig). Regardless of the size of the initial population of gametocytes, the location of the dominant peak of nucleotide diversity does not shift, given a level of starting diversity between the two parasite populations. The parasite diversity within a mosquito does not significantly change between the oocyst stage and the sporozoites found in the salivary glands (see S8 Fig) despite the loss of 80% of sporozoites following oocyst bursting and travel of the sporozoites to the salivary glands. In fact, we observe no loss in the number of genetically distinct parasites from burst oocysts to sporozoites in the salivary glands, so the number of unique sequences does not change (not pictured). However, the nucleotide diversity shifts slightly depending on if the survival of parasites increases or decreases the evenness of populations.

**Fig 8:**
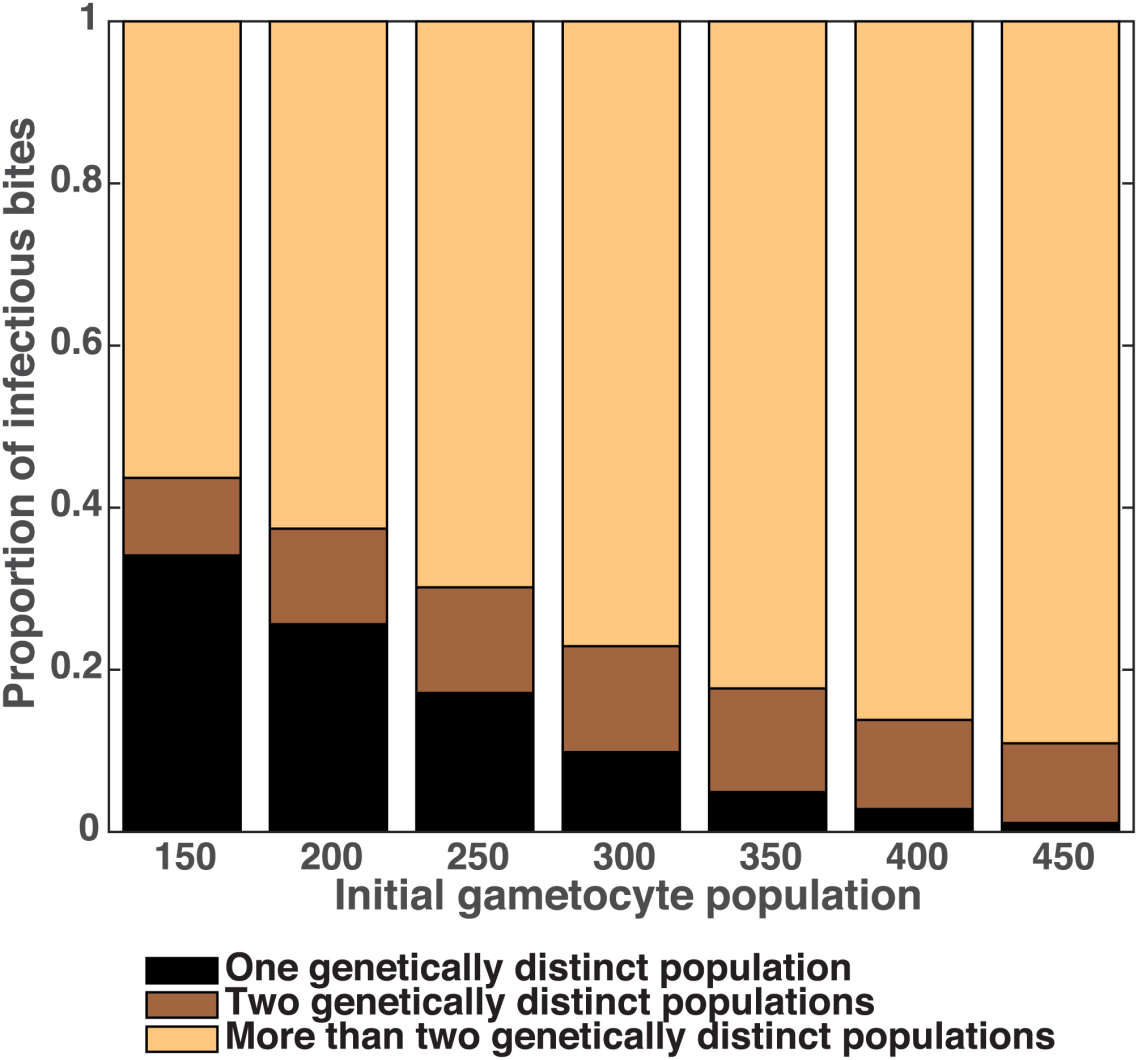
Frequency that multiple genetically distinct parasites are passed in an infectious bite. The fraction of infectious bites that harbor one (black), two (brown) or multiple (beige) parasites with distinct barcode sequences. When the mosquito is infected with a small number of gametocytes, 34% of infectious bites are composed of a single sequence. At larger initial gametocyte numbers the proportion of infectious bites passing only a single genetically distinct parasite population falls to nearly 0. Ten sporozoites were assumed to be present in a single infectious bite.

Fig 8 illustrates that even though only ten sporozoites compose an infectious bite (mean from [48]) the complexity of infection (COI) frequently remains the same or even increases with multiple parasites transmitted simultaneously. With just 150 initial gametocytes from an infection with COI two, nearly 66% of infectious bites contain at least two genetically distinct parasites for onward transmission and over 50% of which increase the COI. As the initial gametocyte number grows nearly all infectious bites contain multiple genetically distinct parasites, maintaining or frequently increasing the COI that is transmitted. Despite observing high levels of COI, several studies have noted that the observed heterozygosity falls below the expected heterozygosity, indicating that novel genotypes may not be generated and transmitted as frequently as predicted [49, 50].

#### Diversity generation using the underlying deterministic model

As the deterministic model, by definition, predicts the same number of gametes, ookinetes, oocysts and sporozoites for a given starting gametocyte population, the resulting diversity observed is severely constrained. When the initial gametocyte population is small (*G*_0_ < 350), the deterministic model predicts fractions of oocysts. In order to compare to results based on life cycle numbers from the CTMC model, we round up to the next highest oocyst number. Thus, exactly four oocysts survive, (one of each type of cross between the two parasite populations), leading to an identical number of unique barcode sequences when *G*_0_ < 350 (see S9 Fig). For larger initial gametocyte numbers, the rounded deterministic model predicts eight oocysts—two of each parasite pairing. This also results in an identical number, albeit higher that what was found with *G*_0_ < 350, of unique barcode sequences—so the nucleotide diversity is identical, regardless of the starting gametocyte number (see S10 Fig). The clear differences between the predicted diversity when using the life cycle numbers from implementation with the underlying stochastic and deterministic models underscores the necessity of considering variation in the parasite development within the mosquito.

## Conclusion

We have introduced a stochastic model of the development of the malaria parasite within the vector to understand the development of diversity in the malaria parasite population. Our results indicate that incorporating variation in the dynamics of life stages of *Plasmodium falciparum* within the mosquito vector can impact the level and diversity of sporozoites produced during the life span of the mosquito. The variation in sporozoite populations predicted, which differ from the implicit assumption of identical sporozoite numbers made by many transmission models [5–8], have potential consequences for the capacity for onward transmission. When modeling diversity generation, we find that the presence of multiple parasite populations within a single mosquito is essential for rapidly increasing diversity in a parasite population. Overall the severe bottlenecks in the life cycle of the parasite reduce population level diversity, but the opportunity for sexual recombination can reintroduce variation when multiple genetically distinct parasite populations are present simultaneously.

Here, we have only considered the dynamics within individual mosquitoes. The next step will be to couple the dynamics within individual mosquitoes with population-level dynamics of transmission, which will allow for inference on infection prevalence. This requires including the human host as well as the vector. Understanding of the potential for diversity generation in the mosquito vector is essential for monitoring and predicting the spread of genetic resistance of the parasite population.

## Supporting information

S1 Fig Diagram of the underlying within-vector parasite dynamics model. Male (*M*) gametes fertilize female (*F*) gametes, creating new zygotes (*Z*) at rate *rMF*. *Z* zygotes progress to the ookinete stage (*E*) at rate *σ*_*z*_*Z*, and *E* ookinetes progress to the oocyst stage (*O*) at rate *σ*_*e*_*E*. Finally, sporoblast formation occurs within the oocysts, leading to *k*(*t*)*O* oocysts rupturing, releasing *n*_0_*k*(*t*)*O* sporozoites, a fraction *p* of which successfully migrate to the mosquito’s salivary glands (depicted in red). All rates are per unit time.
(EPS)

S2 Fig Diagram of the within-vector parasite diversity generation model. Male (*M*) and female (*F*) gametes enter with their unique barcode sequence depicted by gray or white boxes. Each of the 24 positions of the barcode sequence are represented. Adjacent boxes on segments indicate they are found on the same chromosome while ones separated by a small space are on different chromosomes. Only 12 segments are shown as two chromosomes do not have barcode SNPs. The gametes form a new zygote (*Z*) bringing the genetic material of both parasites together. Within the oocyst stage (*O*) the parasites undergo meiosis, briefly forming tetraploid cells where reassortment and recombination of sequences is possible. A cross (x) denotes an example of a position of recombination; in this case all positions to the left of the cross on the same chromosome are swapped between the two barcode sequences. Dashed lines show reassortment of chromosomes, swapping all positions on the chromosome. The new barcode sequences then proliferate asexually within the oocyst, which ultimately rupture producing sporozoites (*S*), each containing one of up to four unique barcode sequences. Pictured here are male and female gametes with maximally different barcode sequences, i.e. all white or all gray boxes, respectively. For simplicity only a single recombination event and a several reassortments are depicted. If the male and female gametes entering are siblings and thus genetically identical, no new genetic combinations are possible and all sporozoites will contain identical barcode sequences. We do not show stages where the parasite diversity is unchanged, such as the ookinete stage.
(EPS)

S3 Fig Oocyst and sporozoite prevalence versus initial gametocyte number. The proportion of mosquitoes harboring oocysts (A) and sporozoites (B) on days 7, 14, and 21 are plotted as a function of the initial gametocyte number *G*_0_ (from *G*_0_ = 10 to 900).
(EPS)

S4 Fig Cumulative formed and ruptured oocysts with step function. The mean cumulative number (on day 21) of oocysts formed (A) and the total number of ruptured oocysts (B) resulting from the 10,000 simulations of the two-parasite population CTMC model (dots) and deterministic model (crosses) is illustrated for initial gametocyte number *G*_0_. Here, the CTMC model utilizes a step rupture function for oocyst bursting, analogous to what is used in the deterministic model. In comparison, Fig 3 uses a continuous time bursting function that is more biologically realistic. Left to right the columns illustrate the results for 0%, 10% and 50% fitness biases, respectively. The stochastic implementation of the life cycle model accurately replicates the mean of the number of formed and burst oocysts.
(EPS)

S5 Fig Comparison of cumulative rupture counts for continuous and step rupture functions. A cumulative count of rupture times using the continuous rupture function (A) and the step rupture function (B) resulting from the 10,000 simulations of the two-parasite population CTMC model. Left to right the columns illustrate the results for 0%, 10% and 50% fitness biases, respectively. The initial gametocyte number is *G*_0_ = 300 in all cases.
(EPS)

S6 Fig Comparison of rupture time frequencies for continuous and step rupture functions. The frequencies of rupture time for the continuous rupture function (A) and the step rupture function (B) resulting from the 10,000 simulations of the two-parasite population CTMC model. Left to right the columns illustrate the results for 0%, 10% and 50% fitness biases, respectively. The initial gametocyte number is *G*_0_ = 300 in all cases.
(EPS)

S7 Fig Composition of nucleotide diversity. (A) Mean nucleotide diversity, *π*, as gametocyte number changes at varying number of starting positions between barcode sequences of the two parasite populations (left to right: 5, 10, 15, 20). When the nucleotide diversity is zero, it is excluded from the mean. 0% fitness bias (blue pluses), 10% fitness bias (red circles) and 50% fitness bias (black crosses) are considered. (B) Histogram of nucleotide diversity, *π*, in the population with 10% bias at varying number of differing of starting positions between barcode sequences of the two parasite populations (left to right: 5, 10, 15, 20). Observe that increasing the initial gametocyte number only affects the distribution of the population but not the diversity at the peak.
(EPS)

S8 Fig Nucleotide diversity comparison between oocysts and sporozoites. Nucleotide diversity, *π*, of the burst oocyst population (x-axis) and sporozoite population in the salivary gland (y-axis). The nucleotide diversity is nearly identical between the two measures, with differences accounted for by changes in population size. Neither appearance nor disappearance of barcode sequences is observed. Points have been subsampled randomly from all initial gametocyte number and biases. A guide (*x* = *y*) line is provided in red for reference.
(EPS)

S9 Fig Number of unique sequences with the deterministic model. (A) The number of unique sequences produced as the number of starting positions differs between barcode sequences of the two parasite populations. Left to right the columns illustrate the results for 0%, 10% and 50% fitness biases, respectively. Observe that for initial gametocyte numbers *G*_0_ < 350 the lines are overlapping. (B) Distribution of the unique sequences when looking at 15 differences in the barcode sequences of the two parasite populations. Left to right the columns illustrate the results for 0%, 10% and 50% fitness biases, respectively. Colors indicate the number of starting gametocytes. Gray bars in (A) show amount of similarity in the two parasite populations for the distributions in (B).
(EPS)

S10 Fig Nucleotide diversity with the deterministic model. Nucleotide diversity, *π*, by differing number of starting positions between barcode sequences of the two parasite populations. Left to right the columns illustrate the results for 0%, 10% and 50% fitness biases, respectively. Colors indicate different initial gametocyte numbers but lines are overlapping.
(EPS)

S1 Appendix Description of simulation method and diversity generation protocol. We present the details of the simulation method for the underlying life-cycle model and the protocol for the generation of parasite diversity. Furthermore, we describe the process of recombination of parasites within-mosquito, and discuss alternative diversity metrics.
(PDF)

S1 File MATLAB code for life-cycle model. MATLAB code for the underlying stochastic life-cycle model.
(PDF)

S2 File MATLAB code for diversity model. MATLAB code for the underlying stochastic diversity model.
(PDF)

S1 Table Two-parasite population CTMC transition matrix. (PDF)

S2 Table Oocyst prevalence and intensity summary statistics. The proportion of oocysts intact on day 7 ranges from approximately 56 to 59%; on day 21 the range is approximately 20-21%.
(PDF)

S3 Table Position of barcode SNPs. The location of the barcode SNPs by chromosome and within each chromosome [41].
(PDF)
