# Supplementary material for: Simulating within-vector generation of the malaria parasite diversity
Source: PLoS One. 2017 May 22;12(5):e0177941. doi: 10.1371/journal.pone.0177941 (PMC5440164; doi:10.1371/journal.pone.0177941)
Supplement: S1 Appendix — We present the details of the simulation method for the underlying life-cycle model and the protocol for the generation of parasite diversity. Furthermore, we describe the process of recombination of parasites within-mosquito, and discuss alternative diversity metrics. (PDF) [file pone.0177941.s011.pdf]

Simulating Within-Vector Generation of the Malaria Parasite Diversity

Lauren M. Childs[1,◕,*], Olivia F. Prosper[2,◕]

1 Department of Mathematics, Virginia Tech, Blacksburg, VA, USA
2 Department of Mathematics, University of Kentucky, Lexington, KY, USA

◕These authors contributed equally to this work.
* lchilds@vt.edu

S1 Appendix: Description of simulation method and diversity generation protocol

Simulating stochastic parasite dynamics model

We modified the Gillespie Algorithm to simulate the CTMC model of parasite dynamics. Our modified Gillespie Algorithm is composed of the following steps:

1. Initialize number of male and female gametes in the blood meal and set all other state variables equal to zero. Set $\Delta t_{\max}$ equal to some small value (we chose $\Delta t_{\max} = 0.1$).

2. Draw 2 numbers y_1 and y_2 from a uniform distribution.

3. Calculate the transition rates for each possible event. For example, using Table 2 for the single parasite population CTMC model, the transition rate for the event 'Death of Male gamete' is aM.

4. Calculate the time to the next event: $\Delta t = -\ln(y_1)/(\text{Sum of all transitions})$. If $\Delta t \leq \Delta t_{\max}$, increase the time by Δt and continue on to steps 5 through 6. Otherwise, increase the time by $\Delta t_{\max}$ and return to step 2.

5. Choose the next event: Create a vector $\vec{r}$ of the transition probabilities (i.e. vector of transition rates divided by sum of all transitions). Then, calculate the cumulative sum of $\vec{r}$ as a vector. Find the last position in this vector that is less than y_2. This position dictates what we choose as the next event in our stochastic realization. The state variables are increased or decreased according to the appropriate transition matrix (see Table 2 for the single parasite population model and S1 Table. for the two parasite population model).

6. If the current time is less than T_{final} (we chose $T_{final} = 21$ days), return to Step 2. Otherwise, stop the simulation here.

The small modification to the Gillespie algorithm, namely comparing Δt to $\Delta t_{\max}$ in Step 4 is necessary to avoid taking unrealistically large time steps between oocyst rupture events when gamete, zygote, and ookinete populations become small. To make sure that $\Delta t_{\max}$ is chosen small enough, we simulated the timing of rupture events with $\Delta t_{\max} = 0.001, 0.01$, and 0.1, and compared the distribution of rupture times and number of ruptured oocysts by day 21. These distributions were quantitatively very similar not pictured, consequently we chose $\Delta t_{\max} = 0.1$ to reduce simulation time. We repeated these simulations 10,000 times, to produce within-vector parasite dynamics for 10,000 mosquitoes, for seven initial gametocyte numbers, G_0, and in the case of the two parasite population model, for three fitness biases between the two parasite populations.

Diversity metrics

Over the course of the parasite life cycle, the within-host diversity shifts: (i) due to bottlenecks moving between humans and mosquitoes, and vice versa, (ii) following oocyst formation due to meiosis and (iii) at the sporozoite formation as only some of the oocysts burst. As a result we must compare between different parasites in the mosquito post-bite and: (i) the burst oocyst population and (ii) the sporozoite population in the salivary glands.

At each time point the population consists of barcode sequences that differ by between zero and 24 positions, p_B. However many of the barcode sequences are identical and can be grouped together. In particular, we aim to measure the diversity in the population of barcode sequences including both the differences in unique barcode sequences and the abundance of these barcode sequences. A major problem in comparing between populations is that they are related by descent, i.e. gametocyte populations are linked to the oocyst and sporozoite populations by fragments of barcode sequence. Furthermore, when there is little diversity in the barcode sequences to begin with there is a maximal amount of diversity that can be achieved even with recombination. In such cases, low absolute diversity is a product of the starting diversity not the potential for adaptation. Ultimately we focused on the nucleotide diversity metric, π, and the number of unique barcode sequences produced. We also considered Hamming distance, average Hamming distance, k-means clustering, hierarchal clustering, Shannon diversity, Simpson diversity and percent of sequences achieved but found them inadequate for our purposes.

Modeling recombination

We built 24 position sequences designed to represent barcode SNP [1] representation of parasite populations. As such, when tracking reassortment and recombination of the parasite genome, we only considered whether these processes altered the SNPs we were following. Of the 24 SNPs, there was one each on chromosomes 2, 4, 5, 8, 9, and 14; two each on chromosomes 1, 6, 10, 12 and 13; and chromosome 7 contained eight [1]. Chromosomes 3 and 12 did not harbor any SNPs, and thus was not considered in the reassortment process. See S3 Table. for the position of the SNPs on each chromosome. A Poisson random variable was selected to determine the number of crossovers per chromosome. A uniform random variable based on the length of the chromosome was used to determine where on the chromosome crossing over would happen. In the case of multiple crossover events, each subsequent crossover event required a uniform random variable to choose which barcode sequence (of the four paired together) for each chromosome and an additional uniform random variable to determine where that crossover event occurred. The position of the crossovers relative to the SNPs would determine if the SNP changed barcode sequences. After recombination, a uniform random variable was chosen for each chromosome to determine whether or not there was reassortment.

Diversity Simulation Protocol

We simulate diversity generation as follows:

1. Randomly generate a single starting parasite barcode sequence of length L, where each position contains one of A characters. Here, we create a sequence of $L = 24$ to emulate the barcode [1], each position of which contains one of two values, thus A=2.

2. Copy the sequence to create a second parasite barcode sequence and switch the value at p_B of the positions, chosen at random without replacement.

3. Copy each of the two barcode sequences to the total number of male and female gametes of each starting parasite population.

4. Select one male and one female gamete with probability based on the fitness bias. These two gametes will join together to form zygotes (and ultimately oocysts).

5. In the oocyst stage, duplicate the male and female barcode sequences and allow recombination and reassortment as described above. Thus, up to four unique barcode sequences can arise as sporozoites from each oocyst. In the case of a single crossover event, two of the barcode sequences will be identical to the parent barcode sequences.

6. Following the underlying deterministic or stochastic model, select the number of oocysts and sporozoites that survive harboring the generated barcode sequences.

7. Calculate diversity on the barcode sequences of the population of ruptured oocysts and sporozoites that successfully traverse to the salivary glands. Determine how many unique barcode sequences are included if 10 sporozoites compose an infectious bite [2].

References

1. Daniels R, Volkman SK, Milner DA, Mahesh N, Neafsey DE, Park DJ, et al. A general SNP-based molecular barcode for *Plasmodium falciparum* identification and tracking. Malaria J. 2008;7(1):223.

2. Medica DL, Sinnis P. Quantitative dynamics of *Plasmodium yoelii* sporozoite transmission by infected anopheline mosquitoes. Infect Immun. 2005; 73(7):4363–4369.
