# Supplementary material for: Simulating within-vector generation of the malaria parasite diversity
Source: PLoS One. 2017 May 22;12(5):e0177941. doi: 10.1371/journal.pone.0177941 (PMC5440164; doi:10.1371/journal.pone.0177941)
Supplement: S1 File — MATLAB code for the underlying stochastic life-cycle model. (PDF) [file pone.0177941.s012.pdf]

S1 File: MATLAB Code for Underlying Parasite Life-Cycle Model

This document includes the MATLAB code, written in MATLAB 2015 b), for the following m-files, along with their decriptions. Prior to running the code, the user should create 3 folders in their working directory titled “TebohData”, “RawData”, and “CTMC_T21_Data”, respectively.

M-file names

1. Create_Parameter_Set.m
2. Teboh_TwoGenotype.m
3. Run_Teboh_TwoGenotype.m
4. RunCTMCModel.m
5. CTMC_T21_Data.m

Note: User only needs to run items (3-5). (1) and (2) are called by the other files.

Code Descriptions

1. *Create_Parameter_Set.m* is a function defining a structure of parameter values. The fitness bias and number of simulations of the CTMC model can be modified here. Files (2) and (4) above call Create_Parameter_Set.m. Note: the number of initial gametocytes can be changed to any natural number when Create_Parameter_Set.m is being called by RunCTMCModel.m, however items (2) and (5) above are designed to run for exactly two genotypes.
2. *Teboh_TwoGenotype.m* is a function requiring as input the initial number of genotype 1 gametocytes G01, genotype 2 gametocytes G02, and total gametocytes G0. This function also solves the deterministic two-genotype model based on the model of Teboh-Ewungkem et al. The model equations are defined as a nested function called Teboh_Mixed.
3. *Run_Teboh_TwoGenotype.m* cycles through the 7 initial gametocyte densities (150, 200,…,450), computes the proportion of these initial gametocytes that are genotype 1 and genotype 2, and evaluates Teboh_TwoGenotype.m for these initial values. Output data will be saved to the folder “TebohData”, and will be named Teboh_Two_Strain_Data_G0=XXX_max_bias=YYY.mat, where XXX is the initial gametocyte density, and YYY is the fitness bias.
4. *RunCTMCModel.m* simulates the stochastic underlying life cycle model (the CTMC model) using a modified Gillespie algorithm as described in the Supplementary Information. Output Data will be saved to the folder “RawData”, and will be named nParasiteGroupsData_G0XXX_max_biasYYY_NumStrainsZZZ, where XXX and YYY are as in item (3) above, and ZZZ is the initial number of genotypes, specified in Create_Parameter_Set.m.
5. *CTMC_T21_Data.m* loads the data in the folder “RawData” and uses it to calculate the initial number of male and female gametes, the total number of oocysts that have ruptured by day 21, and the total number of sporozoites on day 21. Output Data will be saved to the folder “CTMC_T21_Data” and will be named nParasiteGroupsData_G0XXX_max_biasYYY_NumStrains2. This output data will be used as input to the sequence diversity model.

```
function param = Create_Parameter_Set
% Function creates structure of parameter values.  Number of
 genotypes,
% simulations, and max fitness bias can also be modified here.


param.N = 2; % Number of different starting genotypes

param.a = 24/(20/60); % failure rate of male gametocytes (per day)
param.b = 24/(25/60); % failure rate of female gametocytes (per day)
param.r = 0.08; % fertilization of male and female gametes (per day)
param.mu_z = 1; % death rate of zygotes (per day)
param.sigma_z = 24/19; % transformation rate of zygotes (per day)
param.sigma_e = 0.6; % transformation rate of ookinetes (per day)
param.mu_e = 1.4; % death rate of ookinetes (per day)
param.mu_o = 0; % death rate of oocysts (per day)
param.n = 3e3; % number of sporozoites per oocyst
param.p = 0.2; % proportion of sporozoites that make it to salivary
 gland
param.alpha = 0.39; % fraction of male gametes that are viable
param.beta = 1; % fraction of female gametes that are viable
param.eta = 1; % number of female gametes per female gametocyte
param.rho = 8; % number of male gametes per male gametocytes

% Define parameters for bursting rate function:
param.k = 1/7;
param.t0 = 10;

% Select Bias (0, 0.1, 0.5 used in manuscript).
param.max_bias = 0;

% Specify number of simulations of CTMC model to run:
param.NumSim = 10000;
```

Published with MATLAB® R2015b

```
function y=Teboh_TwoGenotype(G01,G02,G0)
%   y=Run_Teboh_Mixed(G0)
%
%   Input: G0 = number of gametocytes
%   Output: time course of gametocytes, zygote, ookinete, oocyst,
 sporozoite
%
%   Calls nested function "Teboh_Mixed".

param = Create_Parameter_Set; % Create paramter set


m = 0.25; % proportion of gametocytes that are male

% ----------

tframe = [0 21]; % run time of simulation

% Calculate initial conditions
percent_male1 = 0.25; % proportion of gametocytes that are male of
 strain 1
percent_male2 = 0.25; % proportion of gametocytes that are male of
 strain 2

alpha = param.alpha;
beta = param.beta;
eta = param.eta;
rho = param.rho;
t0 = param.t0;

max_bias = param.max_bias;

alpha1 = alpha; alpha2 = alpha;
beta1 = beta; beta2 = beta;
eta1 = eta; eta2 = eta;
rho1 = rho; rho2 = rho;

Gf01 = ceil((1 - percent_male1)*G01*eta1*beta1); Gm01 =
 ceil(percent_male1*G01*rho1*alpha1); % Female and Male gamete
 densities, strain 1
Gf02 = ceil((1 - percent_male2)*G02*eta2*beta2); Gm02 =
 ceil(percent_male2*G02*rho2*alpha2); % Female and Male gamete
 densities, strain 2



IC = [Gm01 Gm02 Gf01 Gf02 0 0 0 0 0 0 0 0 0 0 0 0 0 0 0 0 0 0 0 0 0 0
 0 0 0 0]; %initial conditions

[t,y]=ode45(@Teboh_Mixed,tframe,IC,[],param);
filename = ['TebohData/Teboh_Two_Strain_Data_G0='
 num2str(G0) '_max_bias=' num2str(max_bias) '.mat'];
```

```
save(filename,'t','y')

%Plot output variables:
h1 = figure;
subplot(3,2,1)
plot(t,y(:,1:4),'linewidth',2)
FinalizeGraph
xlabel('Time')
ylabel('Gametes')
legend('Male 1', 'Male 2', 'Female 1', 'Female 2')
title('Mixed model')
xlim([0 .1])
subplot(3,2,2)
plot(t,y(:,5:8),'linewidth',2)
FinalizeGraph
xlabel('Time')
ylabel('Zygotes')
legend('11', '12', '21', '22')
subplot(3,2,3)
plot(t,y(:,9:12),'linewidth',2)
FinalizeGraph
xlabel('Time')
ylabel('Ookineetes')
legend('11', '12', '21', '22')
subplot(3,2,4)
plot(t,y(:,13:16),'linewidth',2)
FinalizeGraph
xlabel('Time')
ylabel('Oocysts')
legend('11', '12', '21', '22')
subplot(3,2,5)
plot(t,y(:,17:20),'linewidth',2)
FinalizeGraph
xlabel('Time')
ylabel('Sporozoites')
legend('11', '12', '21', '22')


h2 = figure;
subplot(2,2,1)
plot(t,y(:,21),'linewidth',2)
FinalizeGraph
xlabel('Time')
ylabel('Cumulative Oocyst Count')
subplot(2,2,2)
plot(t,y(:,22),'linewidth',2)
FinalizeGraph
xlabel('Time')
ylabel('# Ruptured Oocysts')
subplot(2,2,3)
plot(t,y(:,22)./y(:,21),'linewidth',2)
FinalizeGraph
xlabel('Time')
ylabel('Ruptured Oocyst Prop.')
```

```
subplot(2,2,4)
plot(t,y(:,20)./y(:,22),'linewidth',2)
FinalizeGraph
xlabel('Time')
ylabel('Sporozoites Per Oocyst')


end

function dy = Teboh_Mixed(t,y,par)
%   dy = Teboh_Mixed(t,y,par)
%
%   Input:  t = time
%           y = state variables
%           par = vector of parameters
%   Output: dy = vector of derivatives

a = par.a;
b = par.b;
r = par.r;
mu_z = par.mu_z;
sigma_z = par.sigma_z;
sigma_e = par.sigma_e;
mu_e = par.mu_e;
mu_o = par.mu_o;
n = par.n;
p = par.p;
max_bias = par.max_bias;
t0 = par.t0;
bias = 1+max_bias;

% For now, set all subgroup parameters equal, except a, b, alpha,
 beta, and the mortality rates:
a1 = a; a2 = a*bias;
b1 = b; b2 = b*bias;

r11 = r;
r12 = r;
r21 = r;
r22 = r;

muz11 = mu_z;
muz22 = mu_z*bias;
muz12 = mean([muz11,muz22]);
muz21 = muz12;

sigmaz11 = sigma_z;
sigmaz12 = sigma_z;
sigmaz21 = sigma_z;
sigmaz22 = sigma_z;

sigmaE11 = sigma_e;
sigmaE12 = sigma_e;
sigmaE21 = sigma_e;
```

```
sigmaE22 = sigma_e;

muE11 = mu_e;
muE22 = mu_e*bias;
muE12 = mean([muE11,muE22]);
muE21 = muE12;

muO11 = mu_o;
muO22 = mu_o*bias;
muO12 = mean([muO11,muO22]);
muO21 = muO12;

n11 = n;
n12 = n;
n21 = n;
n22 = n;

p11 = p;
p12 = p;
p21 = p;
p22 = p;



if t < t0
    k=0; % rate oocysts transform to sporozoites
else
    k=par.k;
end

k11 = k;
k12 = k;
k21 = k;
k22 = k;



M1 = y(1);
M2 = y(2);
F1 = y(3);
F2 = y(4);
Z1 = y(5);
Z2 = y(6);
Z3 = y(7);
Z4 = y(8);
E1 = y(9);
E2 = y(10);
E3 = y(11);
E4 = y(12);
O1 = y(13);
O2 = y(14);
O3 = y(15);
O4 = y(16);
```

```
dM1 = ( - a1*M1 - r11*F1*M1 - r21*F2*M1); % Male gametes
dM2 = ( - a2*M2 - r12*F1*M2 - r22*F2*M2); % Male gametes
dF1 = ( - b1*F1 - r11*F1*M1 - r12*M1*F2); % Female gametes
dF2 = ( - b2*F2 - r12*M1*F2 - r22*F2*M2); % Female gametes
dZ1 = (r11*F1*M1 -muz11*Z1 - sigmaz11*Z1); % Zygotes
dZ2 = (r12*F1*M2 -muz12*Z2 - sigmaz12*Z2); % Zygotes
dZ3 = (r21*F2*M1 -muz21*Z3 - sigmaz21*Z3); % Zygotes
dZ4 = (r22*F2*M2 -muz22*Z4 - sigmaz22*Z4); % Zygotes
dE1 = (sigmaz11*Z1 - muE11*E1 - sigmaE11*E1); % Ookinetes
dE2 = (sigmaz12*Z2 - muE12*E2 - sigmaE12*E2); % Ookinetes
dE3 = (sigmaz21*Z3 - muE21*E3 - sigmaE21*E3); % Ookinetes
dE4 = (sigmaz22*Z4 - muE22*E4 - sigmaE22*E4); % Ookinetes
dO1 = (sigmaE11*E1 - muO11*O1 - k11*O1); % Oocysts
dO2 = (sigmaE12*E2 - muO12*O2 - k12*O2); % Oocysts
dO3 = (sigmaE21*E3 - muO21*O3 - k21*O3); % Oocysts
dO4 = (sigmaE22*E4 - muO22*O4 - k22*O4); % Oocysts
dS1 = (n11*p11*k11*O1); % Sporozoites
dS2 = (n12*p12*k12*O2); % Sporozoites
dS3 = (n21*p21*k21*O3); % Sporozoites
dS4 = (n22*p22*k22*O4); % Sporozoites
dOcount = sigmaE11*E1 + sigmaE12*E2 + sigmaE21*E3 + sigmaE22*E4; %
 Cumulative # Oocysts
dBcount = k11*O1 + k12*O2 + k21*O3 + k22*O4; % Cumulative Number of
 Ruptured Oocysts
dO1count = sigmaE11*E1; % Cumulative # Oocysts by subtype
dO2count = sigmaE12*E2;
dO3count = sigmaE21*E3;
dO4count = sigmaE22*E4;
dB1count = k11*O1; % Cumulative Number of Ruptured Oocysts by subtype
dB2count = k12*O2;
dB3count = k21*O3;
dB4count = k22*O4;


dy = [dM1; dM2; dF1; dF2; dZ1; dZ2; dZ3; dZ4; dE1; dE2; dE3; dE4; dO1;
 dO2; ...
    dO3; dO4; dS1; dS2; dS3; dS4; dOcount; dBcount; dO1count;
 dO2count; dO3count; dO4count;...
    dB1count; dB2count; dB3count; dB4count];

end

function FinalizeGraph()

set(gca,'fontsize',16','fontweight','bold','linewidth',2)
xlim([0 21])
end
```

Published with MATLAB® R2015b

```
% -- Solve Two Genotype ODE model for each initial gametocyte density,
 and
% the max fitness bias defined in Create_Parameter_Set. (Uses Step
 Rupture
% Function).
close all
clear

% Vector of Initial gametocyte densities:
G0_vec = 150:50:450;
% Fraction of G0 of genotype 1:
strain1prop = 0.5;

for ii = 1:length(G0_vec)

   G0 = G0_vec(ii);

    G01 = round(strain1prop*G0);
    G02 = G0 - G01;

    Teboh_TwoGenotype(G01,G02,G0);

end
```

Published with MATLAB® R2015b

```
% -- Simulate CTMC model of within-vector parasite dynamics using
 modified
% Gillespie Algorithm (outlined in Supplementary Materials), with
% continuous rupture function.

close all
clear

SEED = rng('shuffle');% set seed

% Set parameters equal to values defined in Create_Parameter_Set.m:
par = Create_Parameter_Set;

N = par.N;
a = par.a;
b = par.b;
r = repmat(par.r,N,N);
mu_z = par.mu_z;
sigma_z = par.sigma_z;
sigma_e = par.sigma_e;
mu_e = par.mu_e;
mu_o = par.mu_o;
n = par.n;
p = par.p;
alpha = par.alpha;
beta = par.beta;
eta = par.eta;
rho = par.rho;
k = par.k;
max_bias = par.max_bias;
t0 = par.t0;

percent_male = repmat(0.25,N,1); % proportion of gametocytes that are
 male of each genotype

% Define vector of fitness bias for each genotype: (this can be
 modified to consider >3 genotypes)
% For N>3, must define vector of fitness biases.
if N == 1
    bias = 0;
elseif N == 2
    bias = [0,max_bias];
elseif N == 3
    bias = [0, 0.1, 0.5];
end

bias = bias';
a_vec = repmat(a,N,1).*(1 + bias);
b_vec = repmat(b,N,1).*(1 + bias);
alpha_vec = repmat(alpha,N,1).*(1 - bias);
beta_vec = repmat(beta,N,1).*(1 - bias);
```

```
mu_z_vec = repmat(mu_z,N,1).*(1 + bias); % zygote mortality rate for
 zygotes whose parents are of the same genotype
mu_e_vec = repmat(mu_e,N,1).*(1 + bias); % ookinete mortality rate for
 ookinetes " ...
mu_o_vec = repmat(mu_o,N,1).*(1 + bias); % oocyst mortality rate
 oocysts " ...


% Create symmetric matrices of biased parameters.  The (i,j) entry is
 the
% parameter value for a parasite that has a genotype (i) female, and
% genotype (j) male parent.

mu_z_mat = NaN(N,N);
mu_e_mat = NaN(N,N);
mu_o_mat = NaN(N,N);

for i = 1:N;
    for j = 1:N;

        mu_z_mat(i,j) = mean([mu_z_vec(i),mu_z_vec(j)]);
        mu_e_mat(i,j) = mean([mu_e_vec(i),mu_e_vec(j)]);
        mu_o_mat(i,j) = mean([mu_o_vec(i),mu_o_vec(j)]);
    end
end



% ---------------------------------------------------------------------
%
% ---- Run NumSim number of simulations for each value of G0 in G0_vec
 as
% defined in CreateParameterSet.m:

% Number of simulations %
NumSim = par.NumSim;

% Max number of iterations for each simulation %
maxiter = 1e3;

% Vector of Initial gametocyte densities %
G0_vec = 150:50:450;

% Max time (in days)
Tfinal = 21;

% Time-step to increment by if the time to next time-step is greater
 than
% dt.
dt = 0.1;

% Fraction of G0 of each subtype (Nx1 vector):
strainprop = repmat(1/N,N,1);
```

```
TransitionIDs = NaN(NumSim,maxiter);


for ii = 1:length(G0_vec)

    G0 = round(strainprop*G0_vec(ii)) % Vector of initial gametocyte
 densities.  G0(i) is the # of genotype i gametocytes.

    Gf0 = ceil((1 - percent_male).*G0.*eta.*beta) % Female gamete
 densities of each genotype
    Gm0 = ceil(percent_male.*G0.*rho.*alpha) % Male gamete densities
 of each genotype

    Time_data = NaN(maxiter,NumSim); % Initialize Time_data vector.

    Male_data = zeros(N,maxiter,NumSim); % Each row is a different
 genotype; each column is a different time step; each layer is a
 different simulation
    Female_data = zeros(N,maxiter,NumSim); % Same as for Male_data
    Zygote_data = zeros(N,N,maxiter,NumSim); % 4D array:
 Zygote(i,j,k,l) is the # of Zygotes with female parent i, male parent
 j, at timestep k, in simulation l
    Ookinete_data = zeros(N,N,maxiter,NumSim); % "
    Oocyst_data = zeros(N,N,maxiter,NumSim); % "
    Sporozoite_data = zeros(N,N,maxiter,NumSim); % "
    Burst_time = zeros(N,N,maxiter,NumSim); % "


    tic
    for j = 1:NumSim

        Male_data(:,1,j) = Gm0; % Set initial conditions for male and
 female gamete data.
        Female_data(:,1,j) = Gf0; % "


        burst_count(:,:,j) = zeros(N,N); % Initialize burst_count 3-D
 array.

        t(1) = 0;
        time = t(1);

        for i = 2:maxiter



            y1 = rand;
            y2 = rand;

            % State variables
            m = Male_data(:,i-1,j); % Column vector where each row is
 a different genotype, and the elements are the # of Male gametes at
 the previous time-step (t_{i-1}) in simulation j.  So, m(k) is the
 number of genotype-k males at time t_{i-1} in sim j.
```

```
            f = Female_data(:,i-1,j);
            z = Zygote_data(:,:,i-1,j); % z(k,l) = # zygotes with
female-k parent and male-l parent at time t_{i-1} in sim j.
            e = Ookinete_data(:,:,i-1,j);
            o = Oocyst_data(:,:,i-1,j);
            s = Sporozoite_data(:,:,i-1,j);

            % Break if states m through o are extinct:
            state_variables = [m;f;reshape(z,N*N,1);...
                reshape(e,N*N,1);reshape(o,N*N,1)];
            if sum(state_variables(:)) == 0
                LastIter(j) = i-1;
                break
            end

            % Assign data values at current time-step, i, to values at
previous time-step, i-1.
            Male_data(:,i,j) = m;
            Female_data(:,i,j) = f;
            Zygote_data(:,:,i,j) = z;
            Ookinete_data(:,:,i,j) = e;
            Oocyst_data(:,:,i,j) = o;
            Sporozoite_data(:,:,i,j) = s;

            % Define bursting function.
            bursting = 1/(1 + (exp(t0 - t(i-1)))); % probability of
bursting at time 'time'
            % Create vectors/matrices of all possible transitions,
arranged by type: death, mating, maturation, bursting.
            Male_death = a_vec.*m;
            Female_death = b_vec.*f;
            Zygote_death = mu_z_mat.*z;
            Ookinete_death = mu_e_mat.*e;
            Oocyst_death = mu_o_mat.*o;
            Mating = r.*(f*m');
            Zyg_maturation = sigma_z*z;
            Ook_maturation = sigma_e*e;
            Ooc_Bursting = k.*bursting.*o;


            % Calculate all possible transitions:
            transitions =
[Male_death;Female_death;reshape(Zygote_death,N*N,1);...

reshape(Ookinete_death,N*N,1);reshape(Oocyst_death,N*N,1);reshape(Mating,N*N,1);.

reshape(Zyg_maturation,N*N,1);reshape(Ook_maturation,N*N,1);reshape(Ooc_Bursting,


            % Create vector of indices marking different transition
types in
            % the transition vector:
            transition_type_indices =
[0;cumsum([length(Male_death);length(Female_death);length(reshape(Zygote_death,N*
```

```
length(reshape(Ookinete_death,N*N,1));length(reshape(Oocyst_death,N*N,1));length(

length(reshape(Zyg_maturation,N*N,1));length(reshape(Ook_maturation,N*N,1));lengt

            % Calculate vector of transition probabilities %
            total_transitions = sum(transitions);
            trans_probs = transitions/total_transitions;

            % Calculate time of next event.  If time > Tfinal, break!
            stoch_time_step = -log(y1)/(total_transitions);
            time1 = stoch_time_step +t(i-1); % time until next event;
            time2 = t(i-1) + dt;

            if min(time1,time2) > Tfinal

                LastIter(j) = i-1;

                break

            elseif stoch_time_step > dt
                t(i) = time2;
            elseif stoch_time_step <= dt
                t(i) = time1;


                % ---------------------- %
                % Determine next event and update population matrices
%

                choose_transition =
find(cumsum(trans_probs)>y2,1,'first');


                TransitionIDs(j,i) = choose_transition;

                if choose_transition <= transition_type_indices(2);
                    % Male gamete death 1
                    CT = choose_transition -
transition_type_indices(1);
                    Male_data(CT,i,j) = m(CT) - 1;

                elseif choose_transition > transition_type_indices(2)
&& choose_transition <= transition_type_indices(3)
                    % Female gamete death 2
                    CT = choose_transition -
transition_type_indices(2);
                    Female_data(CT,i,j) = f(CT) - 1;

                elseif choose_transition > transition_type_indices(3)
&& choose_transition <= transition_type_indices(4)
                    % Zygote Death 3
                    CT_mat =
reshape(transition_type_indices(3)+1:transition_type_indices(4),N,N);
```

```
                    [ix1,ix2] = find(CT_mat == choose_transition);

                    Zygote_data(ix1,ix2,i,j) = z(ix1,ix2) - 1;

                elseif choose_transition > transition_type_indices(4)
&& choose_transition <= transition_type_indices(5)
                    % Ookinete Death 4
                    CT_mat =
reshape(transition_type_indices(4)+1:transition_type_indices(5),N,N);
                    [ix1,ix2] = find(CT_mat == choose_transition);

                    Ookinete_data(ix1,ix2,i,j) = e(ix1,ix2) - 1;

                elseif choose_transition > transition_type_indices(5)
&& choose_transition <= transition_type_indices(6)
                    % Oocyst Death 5
                    CT_mat =
reshape(transition_type_indices(5)+1:transition_type_indices(6),N,N);
                    [ix1,ix2] = find(CT_mat == choose_transition);

                    Oocyst_data(ix1,ix2,i,j) = o(ix1,ix2) - 1;

                elseif choose_transition > transition_type_indices(6)
&& choose_transition <= transition_type_indices(7)
                    % Mating 6
                    CT_mat =
reshape(transition_type_indices(6)+1:transition_type_indices(7),N,N);
                    [ix1,ix2] = find(CT_mat == choose_transition);

                    Male_data(ix2,i,j) = m(ix2) - 1;
                    Female_data(ix1,i,j) = f(ix1) - 1;
                    Zygote_data(ix1,ix2,i,j) = z(ix1,ix2) + 1;

                elseif choose_transition > transition_type_indices(7)
&& choose_transition <= transition_type_indices(8)
                    % Zygote maturation 7
                    CT_mat =
reshape(transition_type_indices(7)+1:transition_type_indices(8),N,N);
                    [ix1,ix2] = find(CT_mat == choose_transition);

                    Zygote_data(ix1,ix2,i,j) = z(ix1,ix2) - 1;
                    Ookinete_data(ix1,ix2,i,j) = e(ix1,ix2) + 1;

                elseif choose_transition > transition_type_indices(8)
&& choose_transition <= transition_type_indices(9)
                    % Ookinete maturation 8
                    CT_mat =
reshape(transition_type_indices(8)+1:transition_type_indices(9),N,N);
                    [ix1,ix2] = find(CT_mat == choose_transition);

                    Ookinete_data(ix1,ix2,i,j) = e(ix1,ix2) - 1;
                    Oocyst_data(ix1,ix2,i,j) = o(ix1,ix2) + 1;
```

```
                elseif choose_transition > transition_type_indices(9)
&& choose_transition <= transition_type_indices(10)
                    % Oocyst bursting 9
                    CT_mat =
reshape(transition_type_indices(9)+1:transition_type_indices(10),N,N);
                    [ix1,ix2] = find(CT_mat == choose_transition);

                    Oocyst_data(ix1,ix2,i,j) = o(ix1,ix2) - 1;
                    Sporozoite_data(ix1,ix2,i,j) = s(ix1,ix2) +
my_binornd(poissrnd(n),p);
                    Burst_time(ix1,ix2,i,j) = time1;
                    burst_count(ix1,ix2,j) = burst_count(ix1,ix2,j) +
1;

                end

            end

        end

        Time_data(1:length(t),j) = t;

        clear t


    end

    toc

        filename = ['RawData/nParasiteGroupsData_G0' ...
            num2str(G0_vec(ii)) '_max_bias'
num2str(max_bias) '_NumStrains' num2str(N) '.mat'];

save(filename,'Time_data','Male_data','Female_data','Zygote_data',...

 'Ookinete_data','Oocyst_data','Sporozoite_data','Burst_time','burst_count','Tran

end
```

Published with MATLAB® R2015b

```
% Extracts values from the output of RunCTMCModel.m required as input
 for
% sequence diversity model, namely (1) initial number of male and
 female
% gametes, (2) total number of oocysts that have ruptured by day 21,
 and
% (3) the total number of sporozoites present on day 21.

clear
close all

max_bias_vec = [0,.1,0.5]; % Vector of maximum fitness bias
G0_vec = 150:50:450; % Vector of initial gametocyte densities
N = 2; % Initial number of unique genotypes in bloodmeal
T = 21; % Day on which to collect data

for ii = 1:length(G0_vec)

    for jj = 1:length(max_bias_vec)

        max_bias = max_bias_vec(jj);

        % Load CTMC output for max_bias
        filename0 = ['RawData/nParasiteGroupsData_G0'...
            num2str(G0_vec(ii)) '_max_bias' num2str(max_bias)...
            '_NumStrains' num2str(N) '.mat'];
        load(filename0,'Time_data','Female_data','Male_data',...
            'Sporozoite_data','Burst_time')


        NumSim = length(Time_data(1,:));
        T21_index = NaN(1,NumSim);
        checkTimes = NaN(1,NumSim);
        FGam = NaN(N,NumSim);
        MGam = NaN(N,NumSim);
        Sporo = NaN(N,N,NumSim);
        BurstCount = NaN(N,N,NumSim);

        % Determine the last index in Time_data for which...
        % Time_data(:,i) <= T
        for i = 1:NumSim
            T21_index(i) = find(Time_data(:,i) <= T,1,'last');
        end

        %--- Count number of oocyst rupture events up to day T = 21:
        BT_tmp1 = Burst_time>0;
        for i = 1:NumSim
            BurstCount(:,:,i) = sum(BT_tmp1(:,:,1:T21_index(i),i),3);
        end

        % Gamete data, Sporozoite data, Number of oocysts that burst
            % (total by day 21).
```

```
        for i = 1:NumSim
            FGam(:,i) = Female_data(:,1,i);
            MGam(:,i) = Male_data(:,1,i);
            Sporo(:,:,i) = Sporozoite_data(:,:,T21_index(i),i);

        end

        filename = ['CTMC_T21_Data/nParasiteGroupsData_G0'...
            num2str(G0_vec(ii)) '_max_bias' num2str(max_bias)...
            '_NumStrains' num2str(N) '.mat'];
        save(filename,'FGam','MGam','Sporo','BurstCount')

    end

end
```

Published with MATLAB® R2015b
