# Supplementary material for: Simulating within-vector generation of the malaria parasite diversity
Source: PLoS One. 2017 May 22;12(5):e0177941. doi: 10.1371/journal.pone.0177941 (PMC5440164; doi:10.1371/journal.pone.0177941)
Supplement: S2 File — MATLAB code for the underlying stochastic diversity model. (PDF) [file pone.0177941.s013.pdf]

S2 File: MATLAB Code for Diversity Model

This document includes the MATLAB code, written in Matlab 2015a) for the following m-files along with their descriptions. Prior to running the code the user should create two folders "CTMC_T21_Data" and "Replicates". Place files (1) and (2) in the folder "CTMC_T21_Data" and files (3)-(7) in "Replicates".

<u>M-file names</u>

1. LoadRawData_NoBias.m
2. LoadRawData_Bias.m
3. RunWVDBarcode.m
4. Iterate_WVD_Stochastic_HP.m
5. WVDSelection_HP.m
6. Recombination.m
7. Chromo.m

Note: User only needs to run items (1)-(3). (4)-(7) are called by file (3).

<u>Code Descriptions</u>

1. *LoadRawData_NoBias.m* translates the output of the Life Cycle Model and prepares it for input for *RunWVDBarcode.m*. The files should be saved in a folder called "CTMC_T21_Data" and will be named *data_G0XXX_bias0_NumStrains2.mat* where XXX is the initial gametocyte density.
2. *LoadRawData_Bias.m* translates the output of the Life Cycle Model and prepares it for input for *RunWVDBarcode.m*. The files should be saved in a folder called CTMC_T21_Data and will be named *data_G0XXX_biasYYY_NumStrains2.mat* where XXX is the initial gametocyte density and YYY is the bias considered.
3. *RunWVDBarcode.m* is the overarching function to the diversity model using the output from *LoadRawData_NoBias.m* or *LoadRawData_Bias.m* standardly stored in a folder called "CTMC_T21_Data". Output of the diversity model will be written to files *Barcode_G0XXX_biasYYY_NumStrains2.mat* where XXX is the number of initial gametocytes and YYY is the bias considered. This function calls *Iterate_WVD_Stochastic_HP.m*.
4. *Iterate_WVD_Stochastic_HP.m* creates the starting sequences representing each parasite in the initial host and runs replicates given the input data from the life cycle model. This function calls "*WVDSelection_HP.m*.
5. *WVDSelection_HP.m* performs diversity generation model where new sequences are created through recombination and reassortment of sequences in the mosquito host. This function calls *Recombination.m* and *Chromo.m.*
6. *Recombination.m* determines where reassortment and recombination occur between paired chromosomes. Recombination prior to reassortment.
7. *Chromo.m* determines where the barcode SNPs lie in the chromosomes. Information on the locations of the chromosomes is taken from Daniels et al, 2008.

```
function LoadRawData_Bias()

% Converts output of deterministic and stochastic life cycle model

Files = {'nParasiteGroupsData_G0150_max_bias0.1_NumStrains2.mat',  ...
'nParasiteGroupsData_G0150_max_bias0.5_NumStrains2.mat', ...
'nParasiteGroupsData_G0200_max_bias0.1_NumStrains2.mat', ...
'nParasiteGroupsData_G0200_max_bias0.5_NumStrains2.mat', ...
'nParasiteGroupsData_G0250_max_bias0.1_NumStrains2.mat', ...
'nParasiteGroupsData_G0250_max_bias0.5_NumStrains2.mat', ...
'nParasiteGroupsData_G0300_max_bias0.1_NumStrains2.mat', ...
'nParasiteGroupsData_G0300_max_bias0.5_NumStrains2.mat', ...
'nParasiteGroupsData_G0350_max_bias0.1_NumStrains2.mat', ...
'nParasiteGroupsData_G0350_max_bias0.5_NumStrains2.mat', ...
'nParasiteGroupsData_G0400_max_bias0.1_NumStrains2.mat', ...
'nParasiteGroupsData_G0400_max_bias0.5_NumStrains2.mat', ...
'nParasiteGroupsData_G0450_max_bias0.1_NumStrains2.mat', ...
'nParasiteGroupsData_G0450_max_bias0.5_NumStrains2.mat'};
length(Files)

Gams = [150 150 200 200 250 250 300 300 350 350 400 400 450 450];
Gam =
 {'150' '150' '200' '200' '250' '250' '300' '300' '350' '350' '400' '400'
  '450' '450'};
bias = repmat({'0.1' '0.5'},1,7);
NumStrains = repmat([2 2 3],1,7);
NumStrain = {'2' '2' 2' '2' '2' '2' '2' '2' '2' '2' '2' '2' '2' '2' };

for j=1:length(Files)
    load(Files{j})
    G0 = Gams(j);
    Gf0 = FGam(:,1)';
    Gm0 = MGam(:,1)';
    new_burst_count = NaN(length(FGam),NumStrains(j)^2);
    new_SpFinal = NaN(length(FGam),NumStrains(j)^2);
    for k=1:length(FGam)
        if NumStrains(j) ==3
        new_burst_count(k,:) = [BurstCount(1,:,k) BurstCount(2,:,k)
 BurstCount(3,:,k)];
        new_SpFinal(k,:) = [Sporo(1,:,k) Sporo(2,:,k) Sporo(3,:,k)];
        else
        new_burst_count(k,:) = [BurstCount(1,:,k) BurstCount(2,:,k)];
        new_SpFinal(k,:) = [Sporo(1,:,k) Sporo(2,:,k)];
        end
    end
    outfile =
 strcat('data_G0',Gam{j},'_bias',bias{j},'_NumStrains',NumStrain{j},'.mat');
    save(outfile,'G0','Gf0','Gm0','new_burst_count','new_SpFinal')
end
```

Published with MATLAB® R2015b

```
function LoadRawData_NoBias()

% Converts output of deterministic and stochastic life cycle model

Files = {'Teboh_Two_Strain_Data_G0=150_max_bias=0',  ...
'Teboh_Two_Strain_Data_G0=200_max_bias=0',  ...
'Teboh_Two_Strain_Data_G0=250_max_bias=0',  ...
'Teboh_Two_Strain_Data_G0=300_max_bias=0',  ...
'Teboh_Two_Strain_Data_G0=350_max_bias=0',  ...
'Teboh_Two_Strain_Data_G0=400_max_bias=0',  ...
'Teboh_Two_Strain_Data_G0=450_max_bias=0'};
length(Files)

Gams = 150:50:450;
Gam = {'150' '200' '250'  '300'  '350'  '400' '450' };
bias = repmat({'0'},1,7);
NumStrains = repmat(2,1,7);
NumStrain = {'2' '2' '2' '2' '2' '2'  '2' };

for j=1:length(Files)
    load(Files{j})
    G0 = Gams(j);
    Gf0 = y(1,3);
    Gm0 = y(1,1);
    new_burst_count = NaN(1,NumStrains(j)^2);
    new_SpFinal = NaN(1,NumStrains(j)^2);
    for k=1:10
        new_burst_count(k,:) = ceil(y(end,27:30));
        new_SpFinal(k,:) = ceil(y(end,17:20));
    end
    outfile =
 strcat('data_G0',Gam{j},'_bias',bias{j},'_NumStrains',NumStrain{j},'.mat');
    save(outfile,'G0','Gf0','Gm0','new_burst_count','new_SpFinal')
end
```

Published with MATLAB® R2015b

```
function RunWVDBarcode()
% Runs the stochastic model of diversity generation for various
 initial
% gametocyte densities and biases

Input = {'../CTMC_T21_Data/data_G0150_bias0.1_NumStrains2.mat', ...
'../CTMC_T21_Data/data_G0150_bias0.5_NumStrains2.mat', ...
'../CTMC_T21_Data/data_G0200_bias0.1_NumStrains2.mat', ...
'../CTMC_T21_Data/data_G0200_bias0.5_NumStrains2.mat', ...
'../CTMC_T21_Data/data_G0250_bias0.1_NumStrains2.mat', ...
'../CTMC_T21_Data/data_G0250_bias0.5_NumStrains2.mat', ...
'../CTMC_T21_Data/data_G0300_bias0.1_NumStrains2.mat', ...
'../CTMC_T21_Data/data_G0300_bias0.5_NumStrains2.mat', ...
'../CTMC_T21_Data/data_G0350_bias0.1_NumStrains2.mat', ...
'../CTMC_T21_Data/data_G0350_bias0.5_NumStrains2.mat', ...
'../CTMC_T21_Data/data_G0400_bias0.1_NumStrains2.mat', ...
'../CTMC_T21_Data/data_G0400_bias0.5_NumStrains2.mat', ...
'../CTMC_T21_Data/data_G0450_bias0.1_NumStrains2.mat', ...
'../CTMC_T21_Data/data_G0450_bias0.5_NumStrains2.mat'};

Output = {'Barcode_G0150_bias0.1_NumStrains2.mat', ...
'Barcode_G0150_bias0.5_NumStrains2.mat', ...
'Barcode_G0200_bias0.1_NumStrains2.mat', ...
'Barcode_G0200_bias0.5_NumStrains2.mat', ...
'Barcode_G0250_bias0.1_NumStrains2.mat', ...
'Barcode_G0250_bias0.5_NumStrains2.mat', ...
'Barcode_G0300_bias0.1_NumStrains2.mat', ...
'Barcode_G0300_bias0.5_NumStrains2.mat', ...
'Barcode_G0350_bias0.1_NumStrains2.mat', ...
'Barcode_G0350_bias0.5_NumStrains2.mat', ...
'Barcode_G0400_bias0.1_NumStrains2.mat', ...
'Barcode_G0400_bias0.5_NumStrains2.mat', ...
'Barcode_G0450_bias0.1_NumStrains2.mat', ...
'Barcode_G0450_bias0.5_NumStrains2.mat'};
tic
for j=1:length(Output)
    Iterate_WVD_Stochastic_HP(Input{j},Output{j});
    toc
end

end % ends RunWVDBarcorde function


function Iterate_WVD_Stochastic_HP(inputfilename,outputfilename)
% Iterates through replicates of life cycle data
% Called by RunWVDBarcode
rng shuffle

load(inputfilename)
% input file loads G0, Gf0, Gm0, new_SpFinal, new_burst_count

pB = 1:24; % between host differences
```

```
len = 24; % length of barcode

Oo = new_burst_count;
Sp = new_SpFinal;

reps =  length(Oo); % how many replicates

A = 2; % biallelic

tic
count = 1;
countOo = 1;


SumOo = sum(Oo,2);
fullreps = length(find(SumOo)); % how many replicates have oocysts

Gametocytes = NaN(fullreps*2*length(pB),26);
Oocysts = NaN(sum(SumOo)*4*length(pB),27);


cout = Chromo();

piout = NaN(fullreps,24);
pioutS = NaN(fullreps,24);
Noutseq = NaN(fullreps,24);


for j=1:length(pB)
Gam1=randi(A,1,len)-1; % make a template sequence with #A AAs

strainmuts = pB(j); % how many positions to change between strain
mutsid = randperm(len,strainmuts); % where are the positions to change
Gam2 = Gam1;
Gam2(1,mutsid) = mod(Gam1(1,mutsid)+1,2); % template of other strain

Gams = [Gam1; Gam2];

GamSize(1) = Gf0(1)+Gm0(1); % how many of type I into mosquito
GamSize(2) = Gf0(1)+Gm0(1); % how many of type II into mosquito
countdiv = 1;

for i=1:reps
    if SumOo(i)>0 % if oocysts are created
        [Gout, Oout,
 Sout]=WVDSelection_HP(Gams,GamSize,Gf0,Gm0,Oo(i,:),Sp(i,:),len,cout);
        Gametocytes(count:count+1,1:24) = Gout;
        Gametocytes(count:count+1,25) = pB(j);
        Gametocytes(count:count+1,26) = i;
        count = count+2;

        [seq, ~]=size(Oout);

        Oocysts(countOo:countOo+seq-1,1:24) = Oout;
```

```
        Oocysts(countOo:countOo+seq-1,25) = pB(j);
        Oocysts(countOo:countOo+seq-1,26) = i;
        Oocysts(countOo:countOo+seq-1,27) = Sout;
        countOo = countOo + seq;
        Tsporo = sum(Sout);

        [out1, a1, c1] =unique(Oout(:,1:24),'rows');
        [r1, ~] = size(out1);
        x = zeros(r1,1);
        s = zeros(r1,1);
        for j1=1:r1
            x(j1) = length(find(c1==j1))/seq;
            s(j1) = sum((c1==j1).*Sout)/Tsporo;
        end
        pi1 = NaN(r1);
        pi2 = NaN(r1);
        for j1=1:r1
            for j2 = j1+1:r1
                diff = sum(out1(j1,1:24)~=out1(j2,1:24))/24;
                pi1(j1,j2) = 2*x(j1)*x(j2)*diff;
                pi2(j1,j2) = 2*s(j1)*s(j2)*diff;
            end
        end
        piout(countdiv,j) = nansum(pi1(:));
        pioutS(countdiv,j) = nansum(pi2(:));
        Noutseq(countdiv,j) = r1;
        countdiv = countdiv + 1;
    end % ends Oo(i)>0

end  % end i=1:reps
end % ends j=1:length(pB)
save(outputfilename,'Gametocytes','Oocysts','piout','pioutS','Noutseq');
end % ends Iterate_WVD_Stochastic_HP function


function [Gametocytes,
 Oocysts,Sporo]=WVDSelection_HP(Gams,GamSize,Gf,Gm,Oo,Sp,len,cout)
%  Performs diversity generation
%  Called by Iterate_WVD_Stochastic_HP
%  Input:   Gams = sequences of gametocytes
%           GamSize = number of each type of gameotcytes
%           Gf = number of female gametes
%           Gm = number of male gametes
%           Oo = number of oocysts
%           Sp = total number of sporozoites
%           len = length of sequence considered
%           cout = probability of recombination for each chromosome



Oo11 = Oo(1);
Oo12 = Oo(2);
Oo21 = Oo(3);
Oo22 = Oo(4);
```

```
Oo = sum(Oo);

GamA = Gams(1,:);
GamB = Gams(2,:);

Oocyst(Oo).M = NaN(1,len); % initialize male gamete to form oocyst
Oocyst(Oo).F = NaN(1,len); % initialize female gamete to form oocyst
Oocyst(Oo).H = NaN(4,len); % initialize four haploid progeny
Sporo = NaN(Oo*4,1);

j=1;
while j<=Oo
    for k=1:Oo11;
        Oocyst(j).M=GamA; % selected male gamete
        Oocyst(j).F=GamA; % selected female female gamete
        Oocyst(j).H=[GamA;GamA;GamA;GamA];
        Sporo(1:j*4) = round(Sp(1)/(Oo11*4));
        j=j+1;
    end
    for k=1:Oo12;
        Oocyst(j).M=GamB; % selected male gamete
        Oocyst(j).F=GamA; % selected female female gamete
        Oocyst(j).H=[GamB;GamA;GamB;GamA];
        seq = Recombination(cout,Oocyst(j).H); % Find where
 recombination is going to occur
        Oocyst(j).H = seq; % Allow recombination at defined positions
        Sporo((j-1)*4+1:j*4,1) = round(Sp(2)/(Oo12*4));
        j=j+1;
    end
    for k=1:Oo21;
        Oocyst(j).M=GamA; % selected male gamete
        Oocyst(j).F=GamB; % selected female female gamete
        Oocyst(j).H=[GamA;GamB;GamA;GamB];
        seq = Recombination(cout,Oocyst(j).H); % Find where
 recombination is going to occur
        Oocyst(j).H = seq; % Allow recombination at defined positions
        Sporo((j-1)*4+1:j*4,1) = round(Sp(3)/(Oo21*4));
        j=j+1;
    end
    for k=1:Oo22;
        Oocyst(j).M=GamB; % selected male gamete
        Oocyst(j).F=GamB; % selected female female gamete
        Oocyst(j).H=[GamB;GamB;GamB;GamB];
        Sporo((j-1)*4+1:j*4,1) = round(Sp(4)/(Oo22*4));
        j=j+1;
    end
end

Gametocytes = Gams;
AllOo=[];

for j=1:Oo
    AllOo = [AllOo; Oocyst(j).H];
```

```
end

Oocysts = AllOo;


end % ends WVDSelection_HP definition


function seq = Recombination(cout,seqin)
% Recombination Function
% Called by WVDSelection_HP

seq = seqin;

crossover = poissrnd(1,1,12); % find which chromosomes have crossovers
 and how many

% Where do crossovers happen
id1 = histc(sort(rand(1,crossover(1))),cout.c1); % find the location
 of crossovers in chromosome 1
id2 = histc(sort(rand(1,crossover(2))),cout.c2); % find the location
 of crossovers in chromosome 2
id4 = histc(sort(rand(1,crossover(3))),cout.c4); % find the location
 of crossovers in chromosome 4
id5 = histc(sort(rand(1,crossover(4))),cout.c5); % find the location
 of crossovers in chromosome 5
id6 = histc(sort(rand(1,crossover(5))),cout.c6); % find the location
 of crossovers in chromosome 6
id7 = histc(sort(rand(1,crossover(6))),cout.c7); % find the location
 of crossovers in chromosome 7
id8 = histc(sort(rand(1,crossover(7))),cout.c8); % find the location
 of crossovers in chromosome 8
id9 = histc(sort(rand(1,crossover(8))),cout.c9); % find the location
 of crossovers in chromosome 9
id10 = histc(sort(rand(1,crossover(9))),cout.c10); % find the location
 of crossovers in chromosome 10
id11 = histc(sort(rand(1,crossover(10))),cout.c11); % find the
 location of crossovers in chromosome 11
id13 = histc(sort(rand(1,crossover(11))),cout.c13); % find the
 location of crossovers in chromosome 13
id14 = histc(sort(rand(1,crossover(12))),cout.c14); % find the
 location of crossovers in chromosome 14

% Find which paired chromosomes have crossover
% (1=> 1&2, 2=> 2&3, 3=> 3&4, 4=> 4&1)

for j=1:(length(id1)-2)
    for k=1:id1(j)
        loc = randi(4);
        tmp = seq(loc,j:2);
        tmp1 = seq(mod(loc,4)+1,j:2);
        seq(loc,j:2) = tmp1;
        seq(mod(loc,4)+1,j:2) = tmp;
    end
```

```
    end

    for j=1:(length(id2)-2)
        for k=1:id2(j)
            loc = randi(4);
            tmp = seq(loc,3);
            tmp1 = seq(mod(loc,4)+1,3);
            seq(loc,3) = tmp1;
            seq(mod(loc,4)+1,3) = tmp;
        end
    end

    for j=1:(length(id4)-2)
        for k=1:id4(j)
            loc = randi(4);
            tmp = seq(loc,4);
            tmp1 = seq(mod(loc,4)+1,4);
            seq(loc,4) = tmp1;
            seq(mod(loc,4)+1,4) = tmp;
        end
    end

    for j=1:(length(id5)-2)
        for k=1:id5(j)
            loc = randi(4);
            tmp = seq(loc,5);
            tmp1 = seq(mod(loc,4)+1,5);
            seq(loc,5) = tmp1;
            seq(mod(loc,4)+1,5) = tmp;
        end
    end

    for j=1:(length(id6)-2)
        for k=1:id6(j)
            loc = randi(4);
            tmp = seq(loc,j+5:7);
            tmp1 = seq(mod(loc,4)+1,j+5:7);
            seq(loc,j+5:7) = tmp1;
            seq(mod(loc,4)+1,j+5:7) = tmp;
        end
    end

    for j=1:(length(id7)-2)
        for k=1:id7(j)
            loc = randi(4);
            tmp = seq(loc,j+7:15);
            tmp1 = seq(mod(loc,4)+1,j+7:15);
            seq(loc,j+7:15) = tmp1;
            seq(mod(loc,4)+1,j+7:15) = tmp;
        end
    end

    for j=1:(length(id8)-2)
        for k=1:id8(j)
```

```
            loc = randi(4);
            tmp = seq(loc,16);
            tmp1 = seq(mod(loc,4)+1,16);
            seq(loc,16) = tmp1;
            seq(mod(loc,4)+1,16) = tmp;
        end
    end

    for j=1:(length(id9)-2)
        for k=1:id9(j)
            loc = randi(4);
            tmp = seq(loc,17);
            tmp1 = seq(mod(loc,4)+1,17);
            seq(loc,17) = tmp1;
            seq(mod(loc,4)+1,17) = tmp;
        end
    end

    for j=1:(length(id10)-2)
        for k=1:id10(j)
            loc = randi(4);
            tmp = seq(loc,j+17:19);
            tmp1 = seq(mod(loc,4)+1,j+17:19);
            seq(loc,j+17:19) = tmp1;
            seq(mod(loc,4)+1,j+17:19) = tmp;
        end
    end

    for j=1:(length(id11)-2)
        for k=1:id11(j)
            loc = randi(4);
            tmp = seq(loc,j+19:21);
            tmp1 = seq(mod(loc,4)+1,j+19:21);
            seq(loc,j+19:21) = tmp1;
            seq(mod(loc,4)+1,j+19:21) = tmp;
        end
    end

    for j=1:(length(id13)-2)
        for k=1:id13(j)
            loc = randi(4);
            tmp = seq(loc,j+21:23);
            tmp1 = seq(mod(loc,4)+1,j+21:23);
            seq(loc,j+21:23) = tmp1;
            seq(mod(loc,4)+1,j+21:23) = tmp;
        end
    end

    for j=1:(length(id14)-2)
        for k=1:id14(j)
            loc = randi(4);
            tmp = seq(loc,24);
            tmp1 = seq(mod(loc,4)+1,24);
            seq(loc,24) = tmp1;
```

```
            seq(mod(loc,4)+1,24) = tmp;
        end
    end

%%%% REASSORTMENT %%%%%

% chromoid =[1 1 2 4 5 6 6 7 7 7 7 7 7 7 7 8 9 10 10 11 11 13 13 14];

seq(:,1:2) = seq(randperm(4),1:2);
seq(:,3) = seq(randperm(4),3);
seq(:,4) = seq(randperm(4),4);
seq(:,5) = seq(randperm(4),5);
seq(:,6:7) = seq(randperm(4),6:7);
seq(:,8:15) = seq(randperm(4),8:15);
seq(:,16) = seq(randperm(4),16);
seq(:,17) = seq(randperm(4),17);
seq(:,18:19) = seq(randperm(4),18:19);
seq(:,20:21) = seq(randperm(4),20:21);
seq(:,22:23) = seq(randperm(4),22:23);
seq(:,24) = seq(randperm(4),24);


end % ends Recombination function


function cout = Chromo()
% Likelihood of recombination
% Called by WVDSelection_HP

% 2 SNPs on chromo 1
chromo1 = [0; 130573; 539044; 643292];
c1 = (chromo1)/chromo1(end);

% 1 SNP on chromo 2
chromo2 = [0; 842803; 1060087];
c2 = (chromo2)/chromo2(end);

% No SNPs on chromo3

% 1 SNP on chromo 4
chromo4 = [0; 282592; 1204112];
c4 = (chromo4)/chromo4(end);

% 1 SNP on chromo 5
chromo5 = [0; 931601; 1343552];
c5 = (chromo5)/chromo5(end);

% 2 SNPs on chromo 6
chromo6 = [0; 145472; 937750; 1337956];
c6 = (chromo6)/chromo6(end);

% 8 SNPs on chromo 7
chromo7 = [0; 277104; 490877; 545056; 657939; 671839; 683772; 792356;
 1415182; 1415200];%1350452];
```

```
c7 = (chromo7)/chromo7(end);

% 1 SNP on chromo 8
chromo8 = [0; 613716; 1323195];
c8 = (chromo8)/chromo8(end);

% 1 SNP on chromo 9
chromo9 = [0; 634010; 1541723];
c9 = (chromo9)/chromo9(end);

% 2 SNPs on chromo 10
chromo10 = [0; 82376; 1403751; 1694445];
c10 = (chromo10)/chromo10(end);

% 2 SNPs on chromo 11
chromo11 = [0; 117114; 406215; 2035250];
c11 = (chromo11)/chromo11(end);

% No SNPs on chromo 12

% 2 SNPs on chromo 13
chromo13 = [0;158614; 1429265; 2747327];
c13 = (chromo13)/chromo13(end);

% 1 SNP on chromo 14
chromo14 = [0; 755729; 3291006];
c14 = (chromo14)/chromo14(end);

cout.c1 = c1;
cout.c2 = c2;
cout.c4 = c4;
cout.c5 = c5;
cout.c6 = c6;
cout.c7 = c7;
cout.c8 = c8;
cout.c9 = c9;
cout.c10 = c10;
cout.c11 = c11;
cout.c13 = c13;
cout.c14 = c14;
end % ends Chromo function
```

Published with MATLAB® R2015b
