# Supplementary material for: Simulating within-vector generation of the malaria parasite diversity
Source: PLoS One. 2017 May 22;12(5):e0177941. doi: 10.1371/journal.pone.0177941 (PMC5440164; doi:10.1371/journal.pone.0177941)
Supplement: S1 Table — (PDF) [file pone.0177941.s014.pdf]

S1 Table. Two parasite population CTMC transition matrix.

Event	M_1	M_2	F_1	F_2	Z_1	Z_2	Z_3	Z_4	E_1	E_2	E_3	E_4	O_1	O_2	O_3	O_4	S_1	S_2	S_3	S_4
**Death**	-1	0	0	0	0	0	0	0	0	0	0	0	0	0	0	0	0	0	0	0
	0	-1	0	0	0	0	0	0	0	0	0	0	0	0	0	0	0	0	0	0
	0	0	-1	0	0	0	0	0	0	0	0	0	0	0	0	0	0	0	0	0
	0	0	0	-1	0	0	0	0	0	0	0	0	0	0	0	0	0	0	0	0
	0	0	0	0	-1	0	0	0	0	0	0	0	0	0	0	0	0	0	0	0
	0	0	0	0	0	-1	0	0	0	0	0	0	0	0	0	0	0	0	0	0
	0	0	0	0	0	0	-1	0	0	0	0	0	0	0	0	0	0	0	0	0
	0	0	0	0	0	0	0	-1	0	0	0	0	0	0	0	0	0	0	0	0
	0	0	0	0	0	0	0	0	-1	0	0	0	0	0	0	0	0	0	0	0
	0	0	0	0	0	0	0	0	0	-1	0	0	0	0	0	0	0	0	0	0
	0	0	0	0	0	0	0	0	0	0	-1	0	0	0	0	0	0	0	0	0
	0	0	0	0	0	0	0	0	0	0	0	-1	0	0	0	0	0	0	0	0
	0	0	0	0	0	0	0	0	0	0	0	0	-1	0	0	0	0	0	0	0
	0	0	0	0	0	0	0	0	0	0	0	0	0	-1	0	0	0	0	0	0
	0	0	0	0	0	0	0	0	0	0	0	0	0	0	-1	0	0	0	0	0
	0	0	0	0	0	0	0	0	0	0	0	0	0	0	0	-1	0	0	0	0
**Fertiliz-**	-1	0	-1	0	1	0	0	0	0	0	0	0	0	0	0	0	0	0	0	0
**ation**	-1	0	0	-1	0	1	0	0	0	0	0	0	0	0	0	0	0	0	0	0
	0	-1	-1	0	0	0	1	0	0	0	0	0	0	0	0	0	0	0	0	0
	0	-1	0	-1	0	0	0	1	0	0	0	0	0	0	0	0	0	0	0	0
**Stage**	0	0	0	0	-1	0	0	0	1	0	0	0	0	0	0	0	0	0	0	0
**Progress-**	0	0	0	0	0	-1	0	0	0	1	0	0	0	0	0	0	0	0	0	0
**ion**	0	0	0	0	0	0	-1	0	0	0	1	0	0	0	0	0	0	0	0	0
$Z \to E$	0	0	0	0	0	0	0	-1	0	0	0	1	0	0	0	0	0	0	0	0
**Stage**	0	0	0	0	0	0	0	0	-1	0	0	0	1	0	0	0	0	0	0	0
**Progress-**	0	0	0	0	0	0	0	0	0	-1	0	0	0	1	0	0	0	0	0	0
**ion**	0	0	0	0	0	0	0	0	0	0	-1	0	0	0	1	0	0	0	0	0
$E \to O$	0	0	0	0	0	0	0	0	0	0	0	-1	0	0	0	1	0	0	0	0
**Oocyst**	0	0	0	0	0	0	0	0	0	0	0	0	-1	0	0	0	n_1	0	0	0
**Rupture**	0	0	0	0	0	0	0	0	0	0	0	0	0	-1	0	0	0	n_2	0	0
	0	0	0	0	0	0	0	0	0	0	0	0	0	0	-1	0	0	0	n_3	0
	0	0	0	0	0	0	0	0	0	0	0	0	0	0	0	-1	0	0	0	n_4
