# Supplementary material for: Simulating within-vector generation of the malaria parasite diversity
Source: PLoS One. 2017 May 22;12(5):e0177941. doi: 10.1371/journal.pone.0177941 (PMC5440164; doi:10.1371/journal.pone.0177941)
Supplement: S2 Table — The proportion of oocysts intact on day 7 ranges from approximately 56 to 59%; on day 21 the range is approximately 20-21%. (PDF) [file pone.0177941.s015.pdf]

S2 Table. Oocyst prevalence and intensity summary statistics. The proportion of oocysts intact on day 7 ranges from approximately 56 to 59%; on day 21 the range is approximately 20-21%.

Bias	G_0	Mean Intensity	Mean Prevalence	Median	Range
0%		0–1	0–0.7	1	1–5
10%	150	0–1	0–0.8	1	1–4
50%		0–1.2	0–0.8	1	1–4
0%		0–1.3	0–0.9	1	1–5
10%	200	0–1.3	0–0.9	1	1–6
50%		0–1.6	0–0.9	1	1–5
0%		0–2.1	0–1	1	1–6
10%	250	0–2	0–1	1	1–8
50%		0–1.9	0–1	1	1–6
0%		0.2–2.5	0.2–1	1	1–7
10%	300	0.1–2.4	0.1–1	1	1–7
50%		0–2.3	0–1	1	1–7
0%		0.3–3.8	0.2–1	2	1–10
10%	350	0.2–2.8	0.2–1	2	1–9
50%		0–2.9	0–1	1	1–8
0%		0.5–4	0.4–1	2	1–9
10%	400	0.5–3.6	0.4–1	2	1–9
50%		0.1–3.6	0.1–1	2	1–8
0%		1–4.1	0.5–1	2	1–10
10%	450	0.7–4.1	0.4–1	2	1–11
50%		0.2–3.9	0.2–1	2	1–10
