# Supplementary material for: Simulating within-vector generation of the malaria parasite diversity
Source: PLoS One. 2017 May 22;12(5):e0177941. doi: 10.1371/journal.pone.0177941 (PMC5440164; doi:10.1371/journal.pone.0177941)
Supplement: S3 Table — The location of the barcode SNPs by chromosome and within each chromosome [41]. (PDF) [file pone.0177941.s016.pdf]

S3 Table. Position of barcode SNPs. The location of the barcode SNPs by chromosome and within each chromosome (Daniels *et al.* Malaria Journal, 2008).

SNP	Chromosome	Position
1	1	130573
2	1	539044
3	2	842803
4	4	282592
5	5	931601
6	6	145472
7	6	937750
8	7	277104
9	7	490877
10	7	545046
11	7	657939
12	7	671839
13	7	683772
14	7	792356
15	7	1415182
16	8	613716
17	9	634010
18	10	82376
19	10	1403751
20	11	117114
21	11	406215
22	13	158614
23	13	1429265
24	14	755729
